# Exosomes drive ferroptosis by stimulating iron accumulation to inhibit bacterial infection in crustaceans

**DOI:** 10.1016/j.jbc.2023.105463

**Published:** 2023-11-15

**Authors:** Qian Sun, Jiawen Yang, Ming Zhang, Yongsheng Zhang, Hongyu Ma, Ngoc Tuan Tran, Xiuli Chen, Yueling Zhang, Kok-Gan Chan, Shengkang Li

**Affiliations:** 1 Guangdong Provincial Key Laboratory of Marine Biology, Shantou University, Shantou, China; 2 Institute of Marine Sciences, Shantou University, Shantou, China; 3 STU-UMT Joint Shellfish Research Laboratory, Shantou University, Shantou, China; 4 Guangxi Academy of Fishery Sciences, Guangxi Key Laboratory of Aquatic Genetic Breeding and Healthy Aquaculture, Guangxi Nanning, China; 5 Faculty of Science, Division of Genetics and Molecular Biology, Institute of Biological Science, University of Malaya, Kuala Lumpur, Malaysia

**Keywords:** ferroptosis, exosomes, CYP, STEAP4, PPAR, CD36, crustaceans

## Abstract

Ferroptosis, characterized by iron-dependent cell death, has recently emerged as a critical defense mechanism against microbial infections. The present study aims to investigate the involvement of exosomes in the induction of ferroptosis and the inhibition of bacterial infection in crustaceans. Our findings provide compelling evidence for the pivotal role of exosomes in the immune response of crustaceans, wherein they facilitate intracellular iron accumulation and activate the ferroptotic pathways. Using RNA-seq and bioinformatic analysis, we demonstrate that cytochrome P450 (CYP) can effectively trigger ferroptosis. Moreover, by conducting an analysis of exosome cargo proteins, we have identified the participation of six-transmembrane epithelial antigen of prostate 4 in the regulation of hemocyte ferroptotic sensitivity. Subsequent functional investigations unveil that six-transmembrane epithelial antigen of prostate 4 enhances cellular Fe^2+^ levels, thereby triggering Fenton reactions and accelerating CYP-mediated lipid peroxidation, ultimately culminating in ferroptotic cell death. Additionally, the Fe^2+^-dependent CYP catalyzes the conversion of arachidonic acid into 20-hydroxyeicosatetraenoic acid, which activates the peroxisome proliferator-activated receptor. Consequently, the downstream target of peroxisome proliferator-activated receptor, cluster of differentiation 36, promotes intracellular fatty acid accumulation, lipid peroxidation, and ferroptosis. These significant findings shed light on the immune defense mechanisms employed by crustaceans and provide potential strategies for combating bacterial infections in this species.

Ferroptosis is a regulated form of cell death characterized by the accumulation of iron-dependent lipid hydroperoxides to lethal levels (1). It exhibits distinct morphological, biochemical, and genetic features that distinguish it from apoptosis, necrosis, and autophagy (2). Ferroptotic cells commonly display mitochondrial abnormalities and are characterized by two primary biochemical features: iron accumulation (3, 4) and lipid peroxidation (5, 6). Additionally, the overexpression of certain proteins, such as prostaglandin-endoperoxide synthase 2, has been regarded as a biomarker for ferroptosis (7). Iron is indispensable for the execution of ferroptosis, as indicated by the term itself, due to the necessity of iron-dependent oxygenases for lipid peroxidation and the ability of ferrous iron to propagate peroxidation reactions through Fenton chemistry (8).

While initial studies focused on mammalian systems, ferroptosis has also been observed in phylogenetically distant species, including plants, protozoa, and fungi, suggesting that it may be one of the oldest forms of regulated cell death (9). In invertebrates, exposure to Cd induces ferroptosis in fruit flies, leading to growth retardation and behavioral abnormalities (10). Activation of small-conductance calcium-activated K^+^ channels induces a glycolytic shift, enhancing the resilience of neuronal cells against ferroptosis, while vitamin B12 deficiency leads to ferroptosis in *Caenorhabditis elegans* (11, 12). Although ferroptosis has been linked to cancer and other noninfectious diseases, its involvement in modulating host–pathogen interactions remains poorly understood.

Recent studies have revealed that bacterial infections can induce host damage through the activation of ferroptosis (13). Ferroptosis of infected macrophages facilitates the dissemination of *Mycobacterium tuberculosis* by releasing it into the extracellular environment (14). Induction of host cell ferroptosis by *Pseudomonas aeruginosa* can contribute to the progression of the disease (15). Ferroptosis can be induced by *Escherichia coli* in the red blood cells of grass carp, and mediation of ferroptosis by *Aeromonas hydrophila* can lead to brain damage in common carp (16). *A. hydrophila* can also mediate ferroptosis, causing brain damage in common carp (17). Additionally, ferroptosis has been observed to be caused by the intracellular marine pathogen *Edwardsiella piscicida* in a wide range of hosts (18). Similarly, other common pathogenic bacteria such as *Shigella* and *Staphylococcus aureus* exhibit significant ferroptosis characteristics, including lipid peroxidation and iron overload, indicating a close association between their infection and the mechanism of ferroptosis (19). The manipulation of pathogen-associated ferroptosis holds promise as a therapeutic strategy against infectious diseases. However, there is limited understanding regarding ferroptosis in the bacterial infection pathogenesis of crustaceans, and further evidence is required to elucidate the involvement of ferroptosis in the aforementioned physiological and pathological processes.

Exosomes have the capability to transport biological materials to recipient cells, thereby regulating ferroptosis. These small membrane vesicles measure 30 to 150 nm and contain a plethora of complex molecules, including proteins, lipids, and coding or non-coding RNAs. Non-coding RNAs derived from exosomes play a regulatory role in ferroptosis-related proteins (20). Cancer-associated fibroblasts secrete exosomal miR-522 in order to suppress ferroptosis in cancer cells. This is achieved by targeting ALOX15 to hinder the accumulation of lipid-reactive oxygen species (ROS) (21). Additionally, ferritin-containing exosomes are believed to facilitate the extrusion of iron from cells, thereby preventing the initiation of ferroptosis (22). Acting as a medium for intercellular communication, exosomes contribute to the initiation and progression of ferroptosis-mediated diseases. Our previous studies have demonstrated that exosomal miRNAs play a role in the antiviral process and the maintenance of hemolymph microbiota homeostasis in the mud crab *Scylla paramamosain* during *Vibrio parahaemolyticus* infection (23, 24, 25). However, interactions between exosome-mediated ferroptosis and disease pathogenesis and treatment have seldom been investigated.

Hence, in the present study, the effect of ferroptosis through the stimulation of exosomes in bacterial infection was investigated for the first time in crustacean mud crab. The findings revealed that exosomes have the specific ability to enhance iron levels and sensitize hemocytes to ferroptosis, thereby inhibiting the invasion of *V. parahaemolyticus* in mud crabs. Additionally, it was observed that postinfection with *V. parahaemolyticus*, six-transmembrane epithelial antigen of prostate 4 (STEAP4) is densely packed within exosomes and has the potential to catalyze Fe^3+^ conversion into cellular Fe^2+^, resulting in increased iron levels. The presence of Fe^2+^ triggers Fenton reactions, leading to increased uptake of fatty acids (FA) and enhanced conversion of arachidonic acid (AA) into 20-hydroxyeicosatetraenoic acid (20-HETE) through cytochrome P450 (CYP) catalysis. This ultimately regulates the transcription of cluster of differentiation 36 (CD36), resulting in the accumulation of lipid ROS and induction of ferroptosis. These findings offer insights into the influence of exosomes on various critical aspects of ferroptosis that contribute to resistance to the pathogenic infection in crustaceans.

## Results

### Ferroptotic stress emerges in hemocytes after bacterial infection

**Figure 1:**
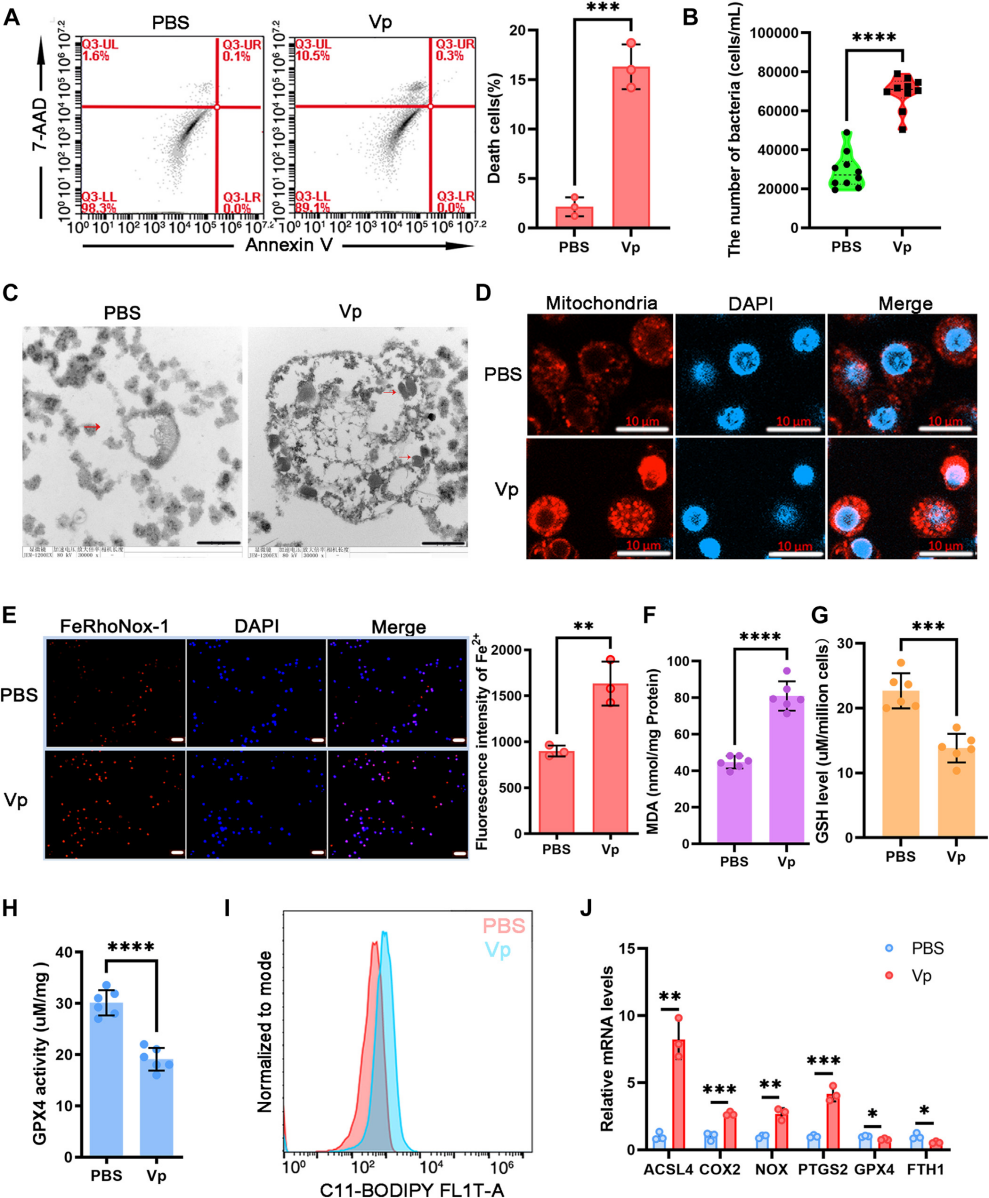
**Ferroptotic stress emerges in hemocytes after bacterial infection.***A*, flow cytometry analysis was performed to evaluate annexin V/7-AAD staining in hemocytes from mud crabs treated with PBS or *Vibrio parahaemolyticus* (about 10^6^ CFU per crab) for 48 h. 7-AAD–positive cells in hemocytes were presented as fold change relative to the PBS-treated mud crabs (data have been normalized to the PBS-treated mud crabs). *B*, bacterial quantification was conducted to determine the total number of bacteria in the hemolymph of mud crabs after treatment with *V. parahaemolyticus* or PBS for 48 h *in vivo*. *C*, TEM was employed to examine the mitochondria in mud crab hemocytes injected with *V. parahaemolyticus* or PBS for 48 h. Scale bars represent 1 μm. *D*, confocal microscopy was utilized to visualize the mitochondria in mud crab hemocytes injected with *V. parahaemolyticus* or PBS for 48 h after staining with MitoTracker Red CMXRos. Scale bars represent 10 μm. *E*, intracellular Fe^2+^ levels of hemocytes treated with *V. parahaemolyticus* for 48 h was detected using FeRhoNox-1 probe and the fluorescence intensity of Fe^2+^ was quantified. Scale bars represent 50 μm. *F*, MDA levels were measured to assess lipid peroxidation in hemocytes. *G*, GSH levels were determined using a GSH assay kit. *H*, GPX4 activity were analyzed using a GPX4 assay kit. *I*, lipid peroxidation was assessed in hemocytes by flow cytometry using the fluorescent probes C11-BODIPY. *J*, qRT-PCR was used to check the expression of ACSL4, COX2, NOX, PTGS2, GPX4, and FTH1 in each sample with β-actin as the control. Bars represent the mean ± SD from three or six independent samples, with at least five crabs per sample. Error bars represent S.D. ∗*p* < 0.05, ∗∗*p* < 0.01, ∗∗∗*p* < 0.001, ∗∗∗∗*p* < 0.0001 (The *p* value was calculated by the *t* test for unpaired samples, and significant differences was accepted when *p* was <0.05.). CFU, colony forming unit; MDA, malondialdehyde; TEM, transmission electron microscopy.

Hemocytes, identified as a vital immune organ in crustaceans, play a crucial role in eradicating bacterial pathogens. Flow cytometry results demonstrated that *V. parahaemolyticus* infection triggers ferroptotic cell death in mud crab hemocytes (Fig. 1*A*). Analysis of bacterial colony-forming units revealed a substantial increase in bacterial concentration in the hemolymph of the *V. parahaemolyticus*–treated group compared to the PBS-treated group (Fig. 1*B*). Additionally, the researchers observed the morphological changes in mitochondria. Examination using transmission electron microscopy (TEM) revealed a reduction in size and loss of cristae in the mitochondria of hemocytes treated with *V. parahaemolyticus* for 48 h (Fig. 1*C*). The distribution and morphology of mitochondria in hemocytes were analyzed using Mitotracker Red CMXRos staining. The study found that *V. parahaemolyticus*–treated hemocytes exhibited clustered distribution of mitochondria in the cytosol, contrasting with the homogeneous filamentous reticular network observed in normal mitochondria (Fig. 1*D*). Moreover, the invasion of *V. parahaemolyticus* led to a significant increase in Fe^2+^ content and the formation of malondialdehyde (MDA) within hemocytes (Fig. 1, *E* and *F*). The GSH content and GPX4 activity decreased in mud crab hemocytes following injection with *V. parahaemolyticus* (Fig. 1, *G* and *H*). Furthermore, hemocytes treated with *V. parahaemolyticus* exhibited elevated lipid peroxide generation, as quantified using C11-BODIPY (Fig. 1*I*). The study also observed a downregulation of GPX4 and FTH1, key regulators of ferroptosis, along with an upregulation of ACSL4, COX2, NOX, and PTGS2 (Fig. 1*J*). These findings suggest the development of ferroptotic stress in hemocytes in response to bacterial infection under *in vivo* conditions.

### Ferroptotic stress contributes to bacterial suppression

**Figure 2:**
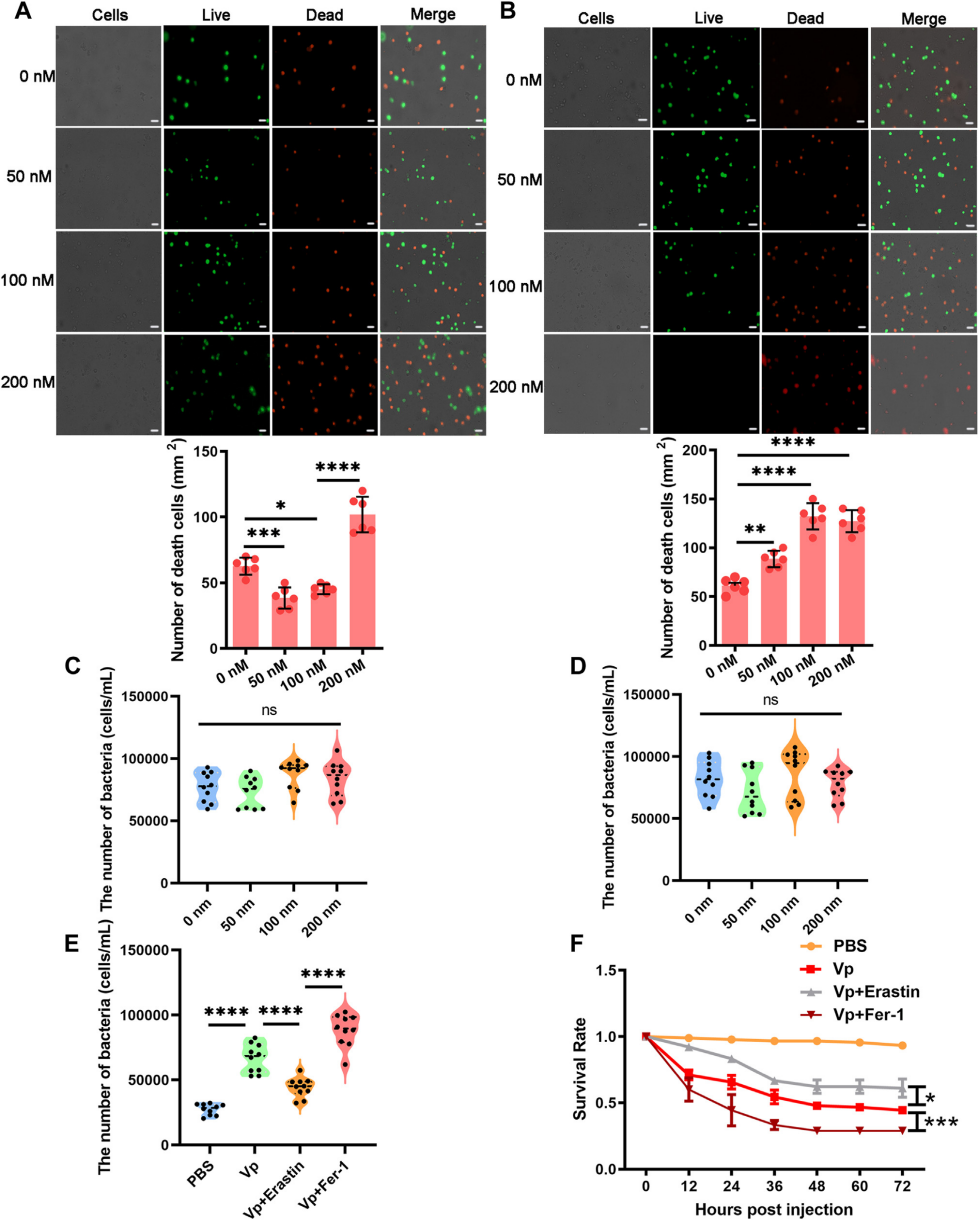
**Ferroptotic stress contributes to bacterial suppression.***A*, the effect of a ferroptosis-inducing compound Erastin on *Vibrio parahaemolyticus*–infected mud crab hemocyte was evaluated using the Calcein-AM/PI Double Stain Kit. Scale bars represent 20 μm. *B*, the effect of a ferroptosis-inhibiting compound Fer-1 on *V. parahaemolyticus*–infected mud crab hemocyte was evaluated using the Calcein-AM/PI Double Stain Kit. Scale bars represent 20 μm. *C* and *D*, Erastin or Fer-1 was incubated with *V. parahaemolyticus*–infected mud crab hemolymph bacteria cultured *in vitro* for 3 h. Bacterial growth was evaluated by measuring the absorbance at 504 nm. *E*, bacterial clearance in mud crabs injected with *V. parahaemolyticus* mixed with Erastin or Fer-1. *F*, the survival rate of mud crabs injected with *V. parahaemolyticus* mixed with Erastin or Fer-1. The bar data are shown as the mean ± S.D. of six independent replicates and analyzed by one-way ANOVA with Tukey’s post hoc test. ∗*p* < 0.05, ∗∗*p* < 0.01, ∗∗∗*p* < 0.001, ∗∗∗∗*p* < 0.0001. PI, propidium iodide.

To investigate the role of hemocytic ferroptosis, hemolymph bacteria collected from *V. parahaemolyticus*–infected mud crabs were treated with either Erastin (a selective ferroptosis promotor) or Fer-1 (Ferrostatin-1, a selective ferroptosis inhibitor) to examine their viability. The mud crab infected with *V. parahaemolyticus* were treated with various concentrations of Erastin or Fer-1, and calcein-AM-propidium iodide (PI) double staining and cell death analysis revealed that treatment with 50 nM and 100 nM Erastin led to a significant reduction in dead cells and an increase in live cells compared to the controls (Fig. 2*A*). Conversely, treatment with 50 nM and 100 nM Fer-1 exhibited the opposite effect on cell death compared to Erastin (Fig. 2*B*). Cultures exposed to increasing concentrations of intact Erastin or Fer-1 (ranging from 0 nM to 200 nM) showed no differences in bacterial growth compared to control cultures (Fig. 2, *C* and *D*). These findings indicate that hemocyte ferroptosis attenuated *V. parahaemolyticus*–induced hemocyte death. To further investigate the impact of Erastin and Fer-1 on bacterial proliferation *in vivo* and the mortality rate, mud crabs were co-injected with *V. parahaemolyticus* along with Erastin or Fer-1. The results demonstrated that Erastin inhibited bacterial proliferation in the hemolymph and increased the cumulative survival of mud crabs, while Fer-1 intensified the damage caused by *V. parahaemolyticus* (Fig. 2, *E* and *F*). In summary, the data suggest that hemocyte ferroptosis play a crucial role in preventing pathogenic invasions.

### CYP is essential for ferroptosis

**Figure 3:**
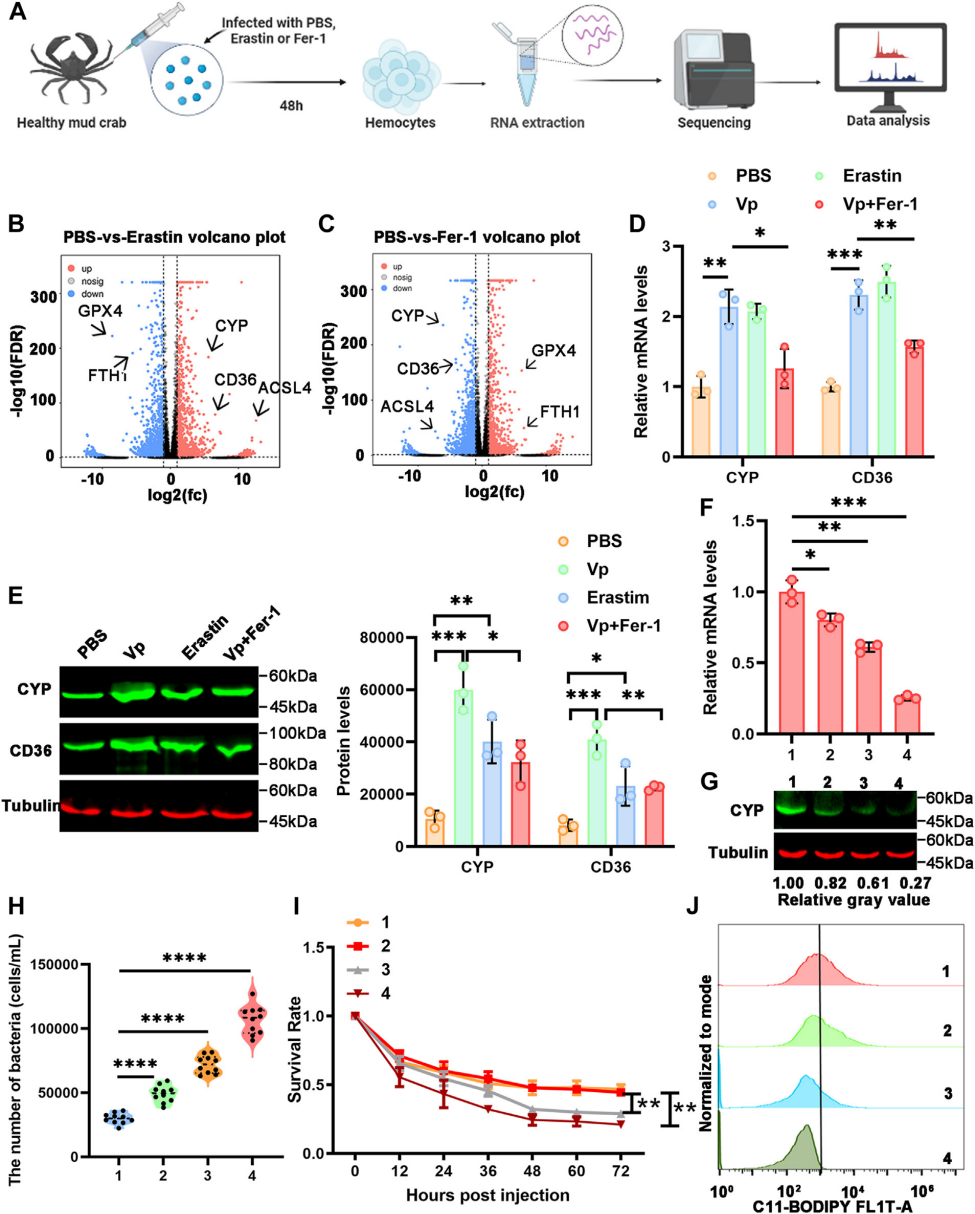
**CYP is essential for ferroptosis.***A*, schematic overview of RNA-seq analysis of hemocyte following treatment with Erastin (100 nM) or Fer-1 (100 nM) for 12 h. *B*, volcano analysis of *top* dysregulated genes in Erastin-treated group. *C*, volcano analysis of top dysregulated genes in Fer-1–treated group. *D*, qRT-PCR analysis of the CYP and CD36 in hemocytes of Erastin, Fer-1, or *Vibrio parahaemolyticus*–injected mud crabs. *E*, Western blot analysis of the CYP and CD36 in hemocytes of Erastin, Fer-1, or *V. parahaemolyticus*–injected mud crabs. *F*, relative CYP expression (qRT-PCR) of mud crab treated with concentration gradient CYP-siRNA was quantified by qRT-PCR. *G*, the CYP protein levels in mud crabs treated with a concentration gradient of CYP-siRNA were measured by Western blot. *H*, hemolymph was collected from mud crabs at day 2 after treatment with *V. parahaemolyticus* and a concentration gradient of CYP-siRNA, and the bacterial counts were determined by plating on agar plates. *I*, the survival rate of mud crabs treated with *V. parahaemolyticus* and a concentration gradient of CYP-siRNA was recorded for 72 h, with three replicate samples and at least ten crabs per sample. *J*, flow cytometry coupled with C11-BODIPY staining was used to determine the levels of lipid peroxides in mud crabs treated with *V. parahaemolyticus* and a concentration gradient of CYP-siRNA. *D* and *E*, two-way ANOVA analysis; Data show mean ± SD; Tukey’s multiple comparisons test; ∗Adjusted *p* < 0.05, ∗∗Adjusted *p* < 0.01, ∗∗∗Adjusted *p* < 0.001; Data from three independent experiments. *F*, *H* and *I*, the data shown represent the mean ± S.D. of three independent replicates and analyzed by one way-ANOVA, ∗*p* < 0.05, ∗∗*p* < 0.01, ∗∗∗*p* < 0.001, ∗∗∗∗*p* < 0.0001. CD36, cluster of differentiation 36.

Although ferroptosis is characterized by lipid peroxidation of organellar and plasma membranes, the possible ferroptosis mechanism in crustaceans remains unclear. To identify potential contributors to ferroptosis, hemocytes from groups treated with PBS, Erastin, and Fer-1 were collected for RNA-seq analysis (Fig. 3*A*). Differentially expressed genes between the Erastin-treated or Fer-1–treated group and the PBS-treated group were screened (Fig. 3, *B* and *C*). Two significantly upregulated mRNAs involved in lipid metabolism, namely *cyp* and *cd36*, were found in the Erastin-treated group, whose expression was significantly downregulated in the Fer-1–treated group. Subsequent quantitative RT-PCR (qRT-PCR) analysis confirmed that the mRNA levels of *cyp* and *cd36* were increased in *V. parahaemolyticus–* or Erastin-treated groups, while they were decreased in the Fer-1–treated groups (Fig. 3*D*). The protein expression of CYP and CD36 showed a consistent pattern with the mRNA levels across the aforementioned groups (Fig. 3*E*). The findings suggest that CYP and CD36 may play an important role in ferroptosis.

As CYP is involved in FA metabolism, specific siRNA was designed to knockdown CYP in hemocytes. The mRNA and protein levels of CYP were assessed in mud crabs injected with graded concentrations of CYP-siRNA (Fig. 3, *F* and *G*). The total number of hemolymph bacteria, survival rate, and lipid peroxidation levels were evaluated in mud crabs injected with different concentrations of CYP-siRNA. The results revealed a negative correlation between CYP expression and the number of total hemolymph bacteria but a positive correlation with the survival rate and lipid peroxidation levels in mud crabs (Fig. 3, *H*–*J*). The results suggest that CYP play a role in ferroptosis and contribute to the resistance against bacterial infection in mud crabs.

### CYP/CD36-dependent fatty acid accumulation dictates cellular sensitivity to ferroptosis

**Figure 4:**
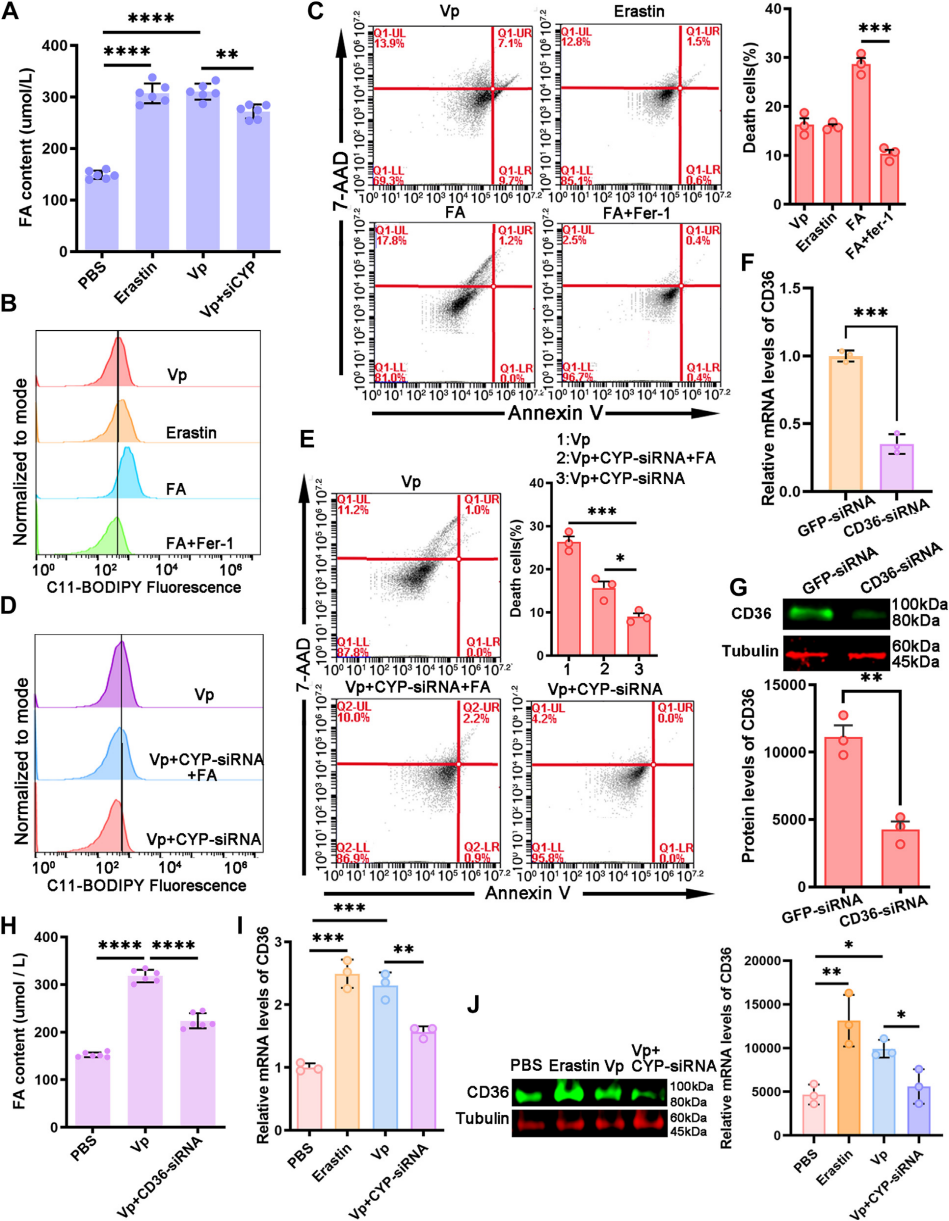
**CYP/CD36-dependent fatty acid accumulation dictates cellular sensitivity to ferroptosis.***A*, an FA uptake assay was performed on mud crabs treated with PBS, Erastin, *Vibrio parahaemolyticus*, or *V. parahaemolyticus* with CYP-siRNA using an FA uptake kit. *B*, flow cytometry coupled with C11-BODIPY staining was used to determine the levels of lipid peroxides in mud crabs treated with *V. parahaemolyticus*, Erastin, FA, or FA with Fer-1. *C*, hemocytes from mud crabs treated with *V. parahaemolyticus*, Erastin, FA, or FA with Fer-1 were subjected to annexin V/7-AAD staining, and the proportion of dead cells (7-AAD-positive) was determined by flow cytometry. *D*, flow cytometry coupled with C11-BODIPY staining was used to determine levels of lipid peroxides in mud crab treated with *V. parahaemolyticus*, *V. parahaemolyticus*, and CYP-siRNA or *V. parahaemolyticus*, CYP-siRNA, and FA. *E*, hemocytes from mud crab treated with *V. parahaemolyticus*, *V. parahaemolyticus*, and CYP-siRNA or *V. parahaemolyticus*, CYP-siRNA, and FA for annexin V/7-AAD staining, and the proportion of 7-AAD–positive cells (dead) was determined by flow cytometry. *F*, the expression of CD36 in crab hemocytes was determined 24 h after injection with CD36-siRNA using qRT-PCR (siGFP served as the control). *G*, Western blot analysis was performed to determine the expression of CD36 in crab hemocytes 24 h after injection with CD36-siRNA (siGFP served as the control). *H*, an FA uptake assay was conducted on mud crabs treated with PBS, *V. parahaemolyticus* or *V. parahaemolyticus* combined with CD36-siRNA. *I*, qRT-PCR was employed to assess the mRNA expression level of CD36 in mud crabs treated with PBS, *V. parahaemolyticus*, Erastin, or *V. parahaemolyticus* combined with CYP-siRNA. *J*, Western blot was used to determine CD36 protein expression level in PBS or mud crab treated with *V. parahaemolyticus*, Erastin, *V. parahaemolyticus* with CYP-siRNA. *A*, *C*, *E*, *H*, *I* and *J*, data represents mean ± SD from three independent repeats with error bars representing S.D. The data were subjected to one-way ANOVA analysis (∗*p* < 0.05, ∗∗*p* < 0.01, ∗∗∗*p* < 0.001, ∗∗∗∗*p* < 0.0001). *F* and *G*, the experiment was performed in three independent replicates, data are shown as the mean ± S.D. Unpaired *t* test. CD36, cluster of differentiation 36; FA, fatty acid.

Quantification of intracellular free FAs was performed, as FA metabolism plays a crucial role in ferroptosis. It was observed that *V. parahaemolyticus* or Erastin treatment induced FA accumulation, which was effectively reversed by the addition of CYP-siRNA (Fig. 4*A*). Furthermore, an increase in lipid peroxidation levels was observed upon the addition of an extra FA mixture, whereas the addition of Fer-1 resulted in the suppression of lipid peroxidation (Fig. 4*B*). *In vitro* studies using hemocytes demonstrated that the addition of Fer-1 deteriorated the cellular sensitivity to FA-induced ferroptosis (Fig. 4*C*). Notably, the knockdown of CYP effectively blocked FA-induced lipid peroxidation and restored ferroptosis insensitivity (Fig. 4, *D* and *E*). These findings strongly suggest that FA be essential for CYP-dependent ferroptosis.

CD36 is a prominent protein involved in FA uptake, particularly in metabolic tissues. Given the influence of Erastin and Fer-1 on CD36 mRNA and protein expression levels, it raised the question that whether CYP-triggered ferroptosis is associated with CD36. In order to investigate the role of CD36 in regulating FA accumulation, a CD36-targeted siRNA was designed to inhibit CD36 expression (Fig. 4, *F* and *G*). Consequently, mud crabs were treated with *V. parahaemolyticus* alone or in combination with CD36-siRNA, and the hemocytes were collected to measure FA content. The results demonstrated that CD36-siRNA inhibited *V. parahaemolyticus*–induced FA uptake by hemocytes (Fig. 4*H*). Furthermore, the promotion of CD36 mRNA expression induced by *V. parahaemolyticus* could be influenced by suppressing CYP expression in hemocytes (Fig. 4, *I* and *J*). These findings indicate that CD36-mediated FA uptake is accountable for CYP-dependent ferroptosis.

### CYP modulates the expression of CD36 through 20-HETE/PPAR pathway

**Figure 5:**
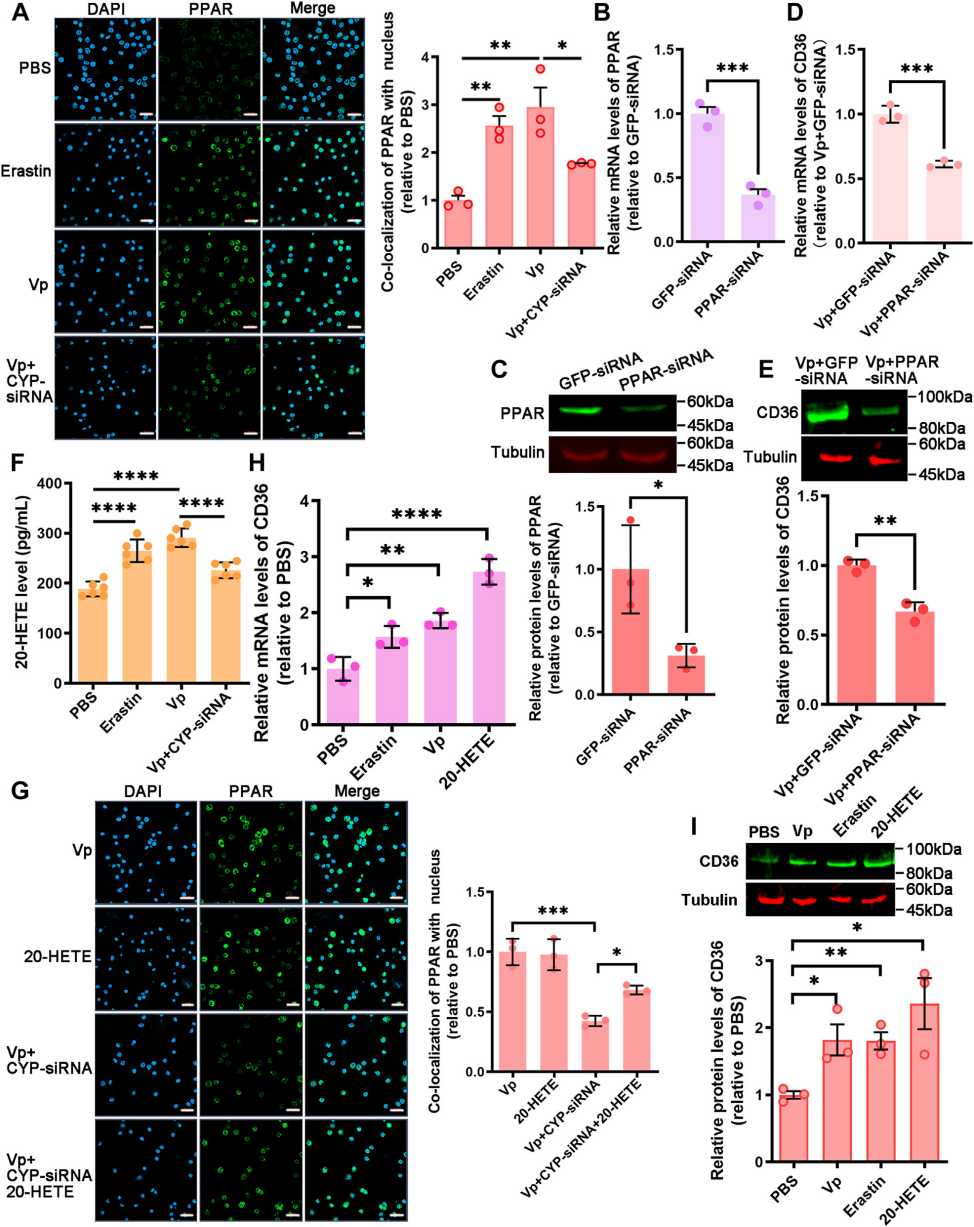
**CYP modulates the expression of CD36 through 20-HETE/PPAR pathway.***A*, translocation of PPAR in hemocytes of CYP-silenced mud crab challenged with *Vibrio parahaemolyticus* compared with that mud crab–injected PBS, Erastin, and *V. parahaemolyticus*. *Green* fluorescence signal indicates the distribution of PPAR in hemocytes, and *blue* shows the nucleus stained with DAPI. Scale bars represent 20 um. *B* and *C*, the expression of PPAR in crab hemocytes was determined 24 h after injection with PPAR-siRNA using qRT-PCR and Western blot (GFP-siRNA served as the control). *D* and *E*, qRT-PCR and Western blot were used to evaluate the relative expression of CD36 in mud crabs treated with *V. parahaemolyticus* combined with GFP-siRNA or *V. parahaemolyticus* combined with PPAR-siRNA. *F*, the levels of 20-HETE were measured in control mud crabs and those treated with *V. parahaemolyticus*, Erastin, or *V. parahaemolyticus* combined with CYP-siRNA. *G*, confocal fluorescence micrographs shows the nuclear PPAR of the *V. parahaemolyticus*, 20-HETE, *V. parahaemolyticus*, and CYP-siRNA or *V. parahaemolyticus*, CYP-siRNA, and 20HETE-treated mud crab. Scale bars represent 20 μm. *H* and *I*, the relative expression of CD36 was determined using qRT-PCR and Western blot in mud crabs treated with PBS, *V. parahaemolyticus*, Erastin, and 20-HETE. *A*, *F*, *G*, *H* and *I*, the data are shown as the mean ± S.D. of three independent replicates and analyzed by one-way ANOVA with Tukey’s post hoc test. ∗*p* < 0.05, ∗∗*p* < 0.01, ∗∗∗*p* < 0.001, ∗∗∗∗*p* < 0.0001. *B*, *C*, *D* and *E*, data are shown as mean ± SD. Student’s *t* tests were performed to obtain statistical significance. CD36, cluster of differentiation 36; HETE, hydroxyeicosatetraenoic acid; PPAR, peroxisome proliferator-activated receptor.

To investigate the regulatory mechanism of CYP on CD36 expression, the nuclear translocation of three nuclear receptors, Liver X receptor, Pregnane X receptor, and PPAR, involved in the regulation of CD36 expression, was examined by Western blot analysis following CYP knockdown. The results indicated that CYP predominantly influenced PPAR nuclear translocation, suggesting that CYP primarily modulates CD36 expression by affecting PPAR nuclear translocation (Fig. S1*F*). Immunofluorescent staining was further used to analyze the distribution of PPAR in nuclear and cytoplasm area. The results demonstrated an increased presence of PPAR in the nucleus of mud crab hemocytes in both the Erastin- and *V. parahaemolyticus*–treated groups. CYP knockdown reduced the PPAR translocation (Fig. S2*A*) and a decreased level was observed in the CYP-siRNA and *V. parahaemolyticus*–treated groups compared with *V. parahaemolyticus*–treated crabs, suggesting that CYP interference hindered the nuclear translocation of PPAR (Fig. 5*A*). To assess whether PPAR controls the expression of CD36, PPAR-targeted siRNA was employed to suppress its expression. This siRNA injection significantly suppressed the expression of PPAR (Fig. 5, *B* and *C*). Subsequently, CD36 expression was assessed through qRT-PCR and Western blot following PPAR-siRNA injection. Hemocytes with PPAR knockdown exhibited lower CD36 expression levelsthan the control groups (comprising GFP-siRNA and *V. parahaemolyticus* injection) (Fig. 5, *D* and *E*). GFP-siRNA, PPAR-siRNA, constructed constitutive active PPAR (26), the prototypical peroxisome proliferator WY-14,643 (27) or WY-14,643, and PPAR-siRNA–treated hemocytes were used to test PPAR involvement in other PPAR target genes other than CD36. Western blot results demonstrated that high levels of PPAR in cells promoted the expression COX-2 and CD36 while suppressing the expression of ICAM-1 (Fig. S1*G*). The findings indicate that CYP promotes FA accumulation by enhancing CD36 upregulation mediated by PPAR.

Further, hemocytes exposed to constructed constitutive active PPAR (PPAR), WY-14,643 or WY-14,643, and PPAR-siRNA were used to detect ferroptotic cell death, GSH and MDA content, GPX4 activity, and expression of genes related to ferroptosis. The results indicated that elevated activation of PPAR in cells can promote iron-dependent cell death (Fig. S2*B*), increase intracellular MDA levels (Fig. S2*E*), and upregulate the expression of genes related to ferroptosis (Fig. S2*F*). Additionally, it leads to a decrease in intracellular GSH levels (Fig. S2*C*) and GPX4 activity (Fig. S2*D*). These experimental evidence confirmed the involvement of PPAR in ferroptosis.

20-HETE is a bioactive lipid produced by the omega-hydroxylation of AA through enzymes belonging to the CYP family. Activation of PPAR can be induced by 20-HETE. The aim of the experiments was to investigate the impact of CYP on the expression of CD36, which is linked to the 20-HETE/PPAR pathway.

Healthy crabs were injected with a concentration gradient of 20-HETE, and a concentration-dependent curve of intracellular 20-HETE was plotted against the measurement of PPAR nuclear translocation (Fig. S1*H*). Hemocytes exhibited increased levels of 20-HETE when treated with *V. parahaemolyticus* or Erastin, but a decrease was observed in the CYP-siRNA–treated group (Fig. 5*F*). Immunostaining analysis demonstrated that *V. parahaemolyticus* or 20-HETE could upregulate PPAR content in the nuclear area. In addition, PPAR was less translocated in CYP knockdown and then rescued *via* 20-HETE addition (Fig. 5*G*). 20-HETE can only be metabolized from AA by the CYP pathway. These results showed that 20-HETE produced by the CYP pathway after *V. parahaemolyticus* infection was sufficient to activate PPAR. Consequently, the expression levels of CD36 were assessed in mud crabs injected with *V. parahaemolyticus*, Erastin, or 20-HETE, with the 20-HETE treatment yielding similar results to the Erastin and *V. parahaemolyticus*–treated groups (Fig. 5, *H* and *I*). The results confirm that CYP-induced CD36 expression in hemocytes is mediated by the 20-HETE/PPAR pathway.

### CYP-dependent metabolites of AA navigate cells to ferroptosis

**Figure 6:**
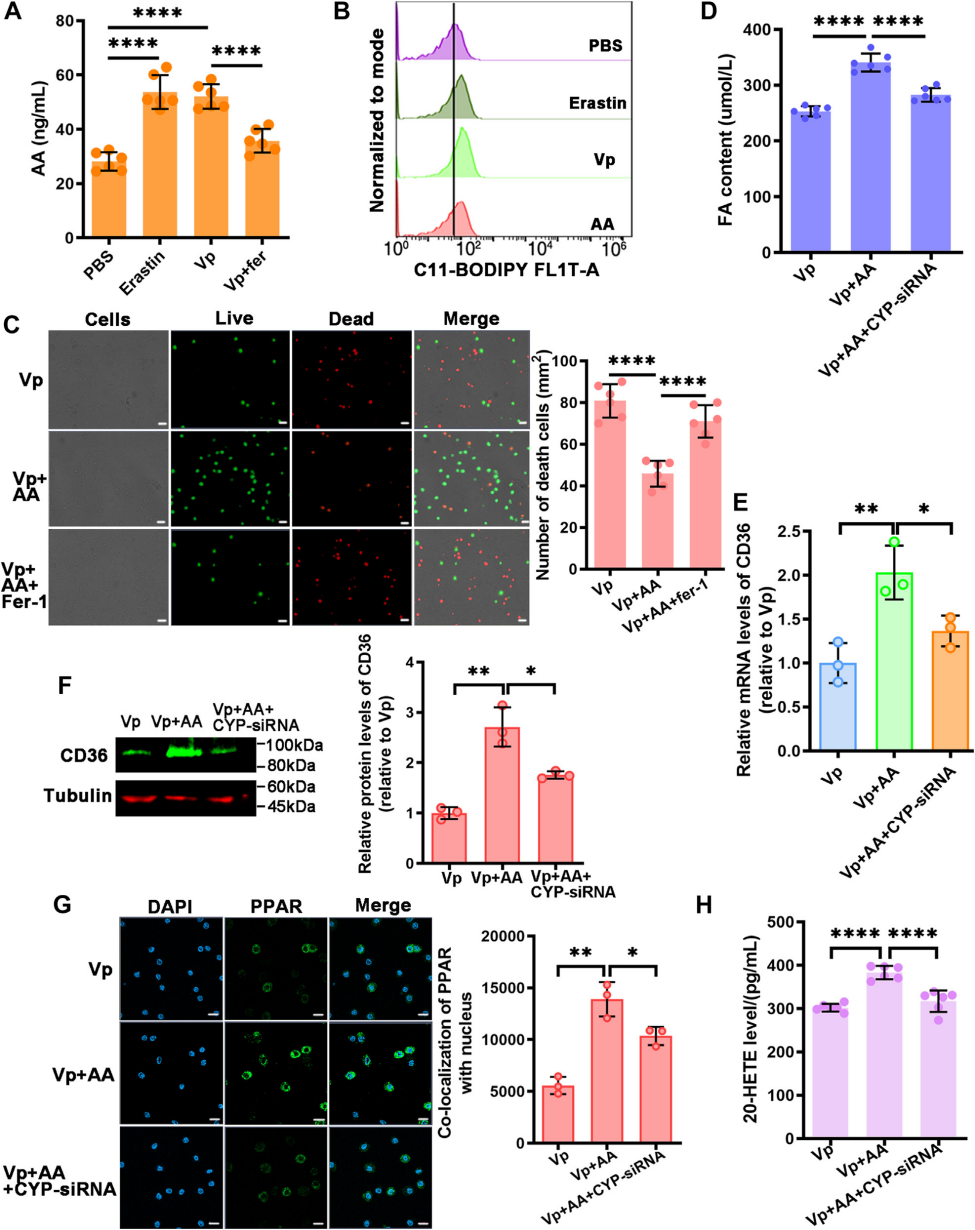
**CYP-dependent metabolites of AA navigate cells to ferroptosis.***A*, hemocytes were injected with PBS, Erastin, *Vibrio parahaemolyticus*, or *V. parahaemolyticus* and Fer-1 before analyzed for AA levels. *B*, lipid peroxidation levels were tested using C11-BODIPY in mud crabs treated with PBS, Erastin, *V. parahaemolyticus*, or AA. *C*, the viability of hemocytes treated with *V. parahaemolyticus*, AA or *V. parahaemolyticus*, AA and Fer-1 was assessed using the Calcein-AM/PI Double Stain Kit. Scale bars represent 20 μm. *D*, the FA content in hemocytes was detected in mud crabs treated with *V. parahaemolyticus*, *V. parahaemolyticus* and AA, or *V. parahaemolyticus*, AA, and CYP-siRNA. *E* and *F*, qRT-PCR and Western blot was used to determine the expression of CD36 in mud crabs treated with *V. parahaemolyticus*, *V. parahaemolyticus* and AA, or *V. parahaemolyticus*, AA, and CYP-siRNA. *G*, the translocation of PPAR in hemocytes of mud crabs treated with *V. parahaemolyticus*, *V. parahaemolyticus* and AA, or *V. parahaemolyticus*, AA, and CYP-siRNA was analyzed by immunofluorescence assay. Scale bars represent 10 μm. *H*, the levels of 20-HETE were measured in controls and those treated with *V. parahaemolyticus*, *V. parahaemolyticus* and AA, or *V. parahaemolyticus*, AA, and CYP-siRNA. Data represent mean ± SD from three independent repeats with error bars representing S.D. The data were subjected to one-way ANOVA analysis (∗*p* < 0.05, ∗∗*p* < 0.01, ∗∗∗∗*p* < 0.0001). CD36, cluster of differentiation 36; HETE, hydroxyeicosatetraenoic acid; FA, fatty acid; PI, propidium iodide; PPAR, peroxisome proliferator-activated receptor.

AA plays a crucial role in peroxidation associated with ferroptosis in the plasma membrane. The CYP enzyme families produce 20-HETE as a metabolite of AA. The involvement of the CYP enzyme in an AA-20-HETE–dependent pathway in ferroptosis was investigated. The AA levels in hemocytes were measured in mud crabs treated with PBS, Erastin, *V. parahaemolyticus* alone, or in combination with Fer-1 infection. Consistent with expectations, Erastin and *V. parahaemolyticus* elevated AA levels in the hemocytes, while Fer-1 reversed the *V. parahaemolyticus*–induced increase in AA contents (Fig. 6*A*). Ultimately, AA induced lipid ROS production and enhanced cell viability following *V. parahaemolyticus* infection (Fig. 6, *B* and *C*).

To determine the involvement of CYP in the ferroptotic cell death pathway, specifically in the increased production of 20-HETE through AA induction, the FA content of hemocytes was assessed (Fig. 6*D*). Knocking down CYP using siRNA prevented the AA-induced increase in FA and CD36 expression in hemocytes (Fig. 6, *E* and *F*). Subsequently, the relocation of PPAR from the cytoplasm to the nucleus was measured, and it was observed that CYP-siRNA decreased the nuclear presence of PPAR induced by AA (Fig. 6*G*). Additionally, the accumulation of AA-induced 20-HETE was inhibited by CYP-siRNA (Fig. 6*H*). These results provide evidence that CYP enhances the production of 20-HETE by metabolizing AA, thereby promoting ferroptosis.

### Exosome enhances hemocyte ferroptosis

**Figure 7:**
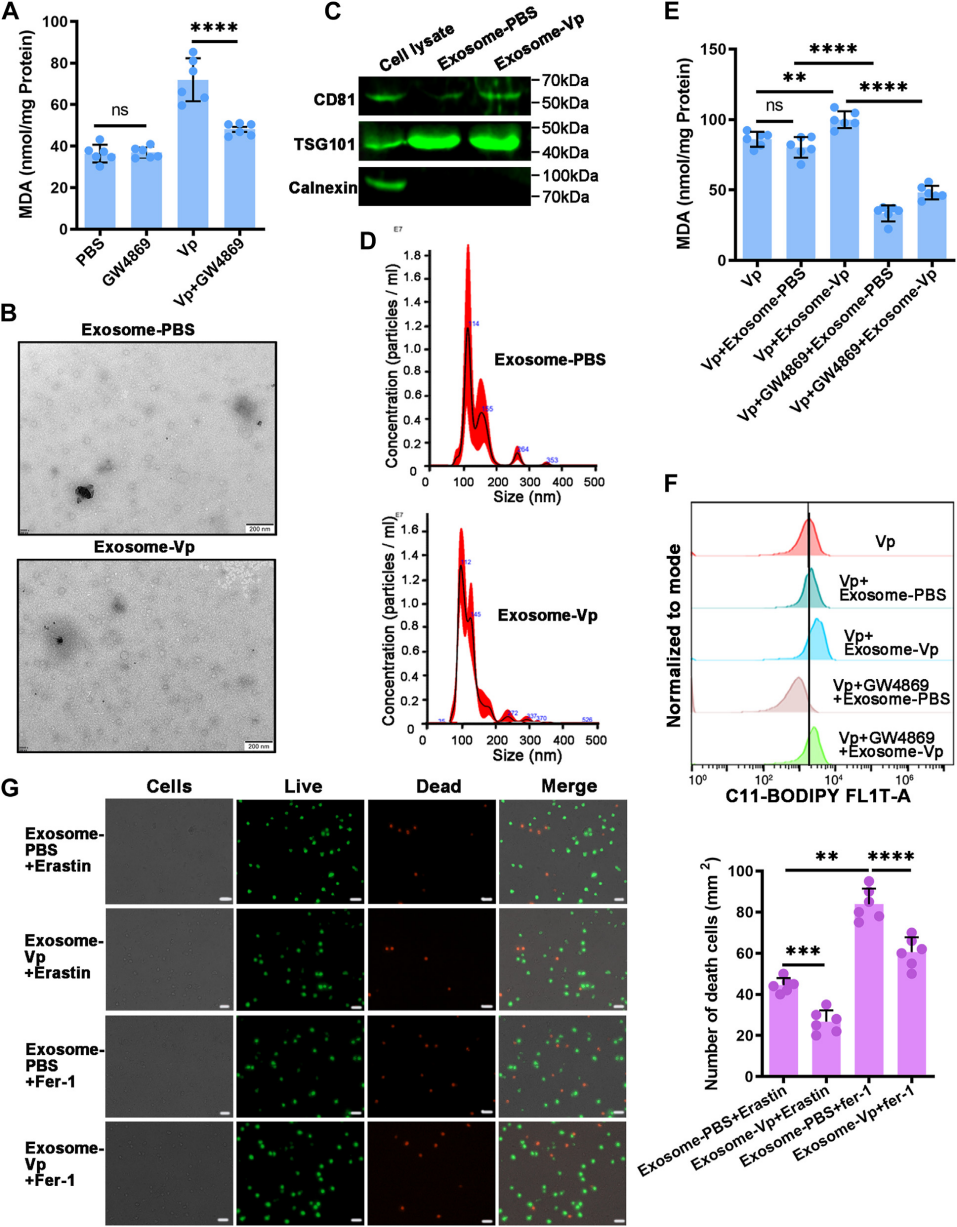
**Exosome enhances hemocyte ferroptosis.***A*, the MDA assay was utilized to quantify lipid peroxidation in the hemocytes of mud crabs treated with PBS, GW4869 and *Vibrio parahaemolyticus* alone, or *V. parahaemolyticus* in combination with GW4869. *B*–*D*, exosomes isolated from mud crabs injected with PBS and *V. parahaemolyticus* were detected by electron microscopy, Western blot analysis of exosomal protein markers (CD81 and TSG101) and cytoplasmic marker (Calnexin, negative control) in the cell lysate and exosomes and Nano-Sight particle tracking analysis. Scale bars represent 200 nm. (Vp: *V. parahaemolyticus*, exosome-Vp/exosome-PBS: exosomes isolated from the hemolymph of crabs challenged with *V. parahaemolyticus*/PBS, respectively). *E*, lipid peroxidation of *V. parahaemolyticus*–treated mud crabs in the presence of exosome-Vp/exosome-PBS or exosome-Vp/exosome-PBS along with GW4869 was detected using the MDA assay. *F*, flow cytometry, coupled with C11-BODIPY staining, was employed to determine the levels of lipid peroxides in the hemocytes of *V. parahaemolyticus*–treated mud crabs in the presence of exosome-Vp/exosome-PBS or exosome-Vp/exosome-PBS along with GW4869. *G*, the Calcein-AM/PI Double Stain Kit was used to evaluate the effect of exosome-Vp/exosome-PBS, along with Erastin or Fer-1, on cell death. Scale bars represent 20 μm. *A*, *E*, and *G*, the data shown represent the mean ± S.D. of three independent replicates and analyzed by one way-ANOVA, ∗*p* < 0.05, ∗∗*p* < 0.01, ∗∗∗*p* < 0.001, ∗∗∗∗*p* < 0.0001. MDA, malondialdehyde; PI, propidium iodide.

With inspiration from the antibacterial properties of exosome-Vp (exosomes collected from *V. parahaemolyticus*–infected mud crab hemolymph), the effects of exosome-Vp on *V. parahaemolyticus*–induced ferroptosis were investigated in this study. GW4869, a potent inhibitor of sphingomyelinase crucial for exosome formation, was employed to suppress the release of exosomes in mud crabs pretreated with *V. parahaemolyticus* (28). GW4869 itself had no effect on MDA levels. Intriguingly, GW4869 was found to alleviate the *V. parahaemolyticus*–enhanced MDA levels (Fig. 7*A*). This observation suggests that exosome-Vp may contribute to the hemocyte ferroptosis induced by *V. parahaemolyticus*.

To investigate the involvement of exosome-Vp in hemocyte ferroptosis, mud crabs were injected with PBS (referred to as exosome-PBS). The release of exosomes from the hemolymph was confirmed through nanoparticle tracking analysis, TEM, and immunoblotting. TEM analysis, conducted after negative staining with uranyl acetate, revealed that the particles exhibited a characteristic cup-shaped morphology and had an approximate size of 100 nm (Fig. 7*B*). Immunoblotting of the particles confirmed the expression of exosome surface protein markers CD63 and CD81. This was further corroborated by the negative control Calnexin, which is expressed in the endoplasmic reticulum membrane (Fig. 7*C*). Nanoparticle tracking analysis results revealed that the majority of the particles were around 100 nm in size, but there were also some larger vesicles present (Fig. 7*D*). These findings confirm the successful isolation of exosomes from mud crabs challenged with either *V. parahaemolyticus* or PBS.

To investigate the regulatory role of exosome-Vp in ferroptosis, mud crabs infected with *V. parahaemolyticus* were treated with exosomes (exosome-Vp or exosome-PBS) either alone or in combination with GW4869. Subsequently, lipid peroxidation levels were measured across the groups. Vp+Exosome-Vp treatment resulted in higher lipid peroxidation than Vp+Exosome-PBS treatment. Furthermore, GW4869 effectively attenuated lipid peroxidation induced by exosome-Vp (Fig. 7, *E* and *F*). Calcein-AM-PI double staining revealed that Erastin treatment significantly decreased cell death and increased the number of viable cells in mud crabs treated with exosome-Vp. Conversely, Fer-1 completely negated the inhibitory effect of exosome-Vp on cell death (Fig. 7*G*). These results showed that exosome-Vp promotes ferroptosis in hemocytes to protect mud crabs from *V. parahaemolyticus* invasion.

### Exosome promotes ferroptosis *via* CYP–CD36 axis

**Figure 8:**
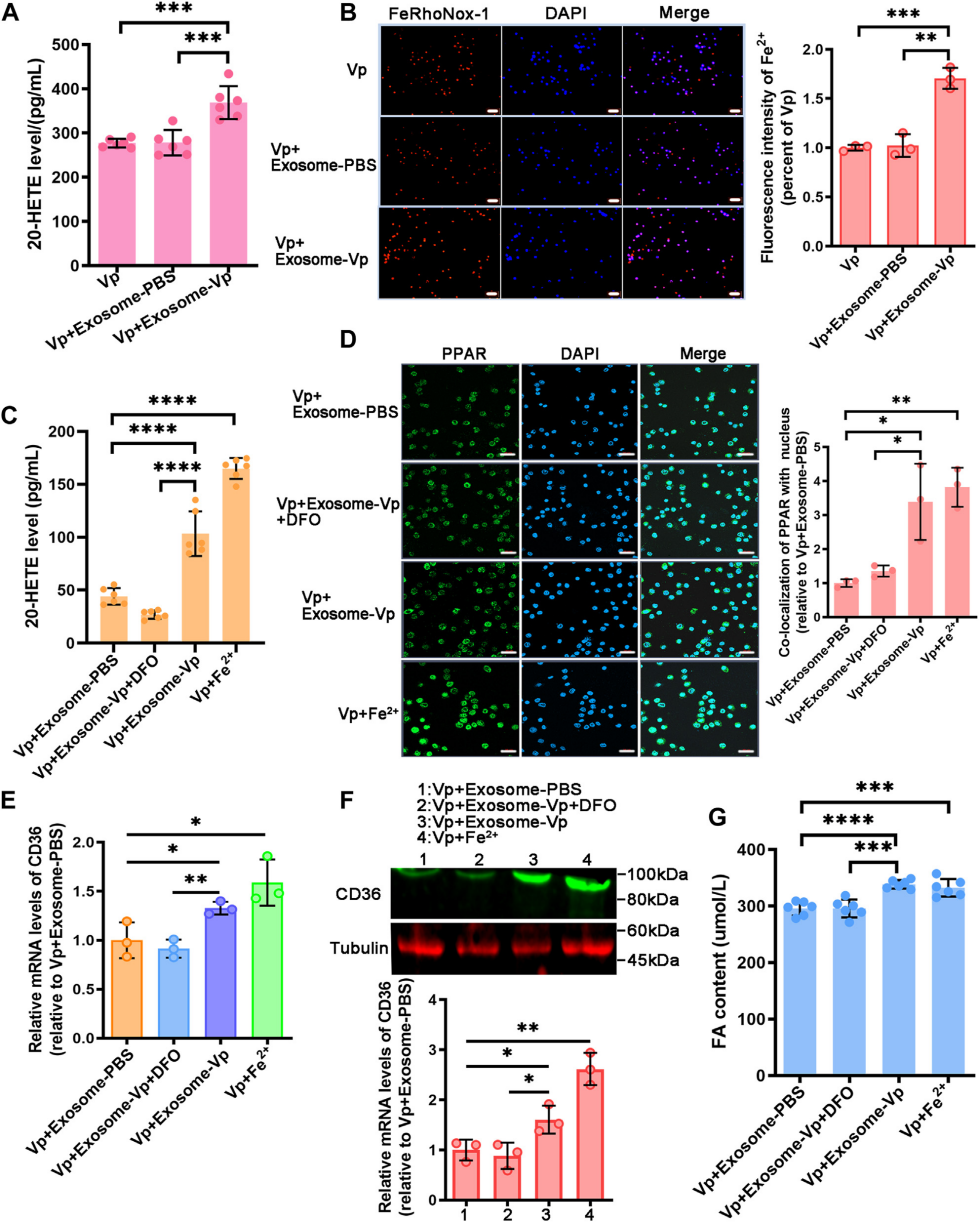
**Exosome promotes ferroptosis *via* CYP-CD36 axis.***A*, the level of 20-HETE was measured in mud crabs injected with *Vibrio parahaemolyticus*, *V. parahaemolyticus* and exosome-PBS, or *V. parahaemolyticus* and exosome-Vp. *B*, FeRhoNox-1 staining, coupled with confocal microscopy, visualized the intracellular Fe^2+^ levels after treatment with *V. parahaemolyticus*, *V. parahaemolyticus* and exosome-PBS, or *V. parahaemolyticus* and exosome-Vp. Scale bars represent 50 μm. *C*, cultured hemocytes from *V. parahaemolyticus*–infected mud crab were treated with exosome-PBS, exosome-Vp, exosome-Vp and DFO, or Fe^2+^, and the 20-HETE levels were measured in each group. *D*, immunohistochemistry showing the PPAR in each group. Scale bars represent, 20 μm. *E* and *F*, CD36 expression was determined by qRT-PCR and Western blot in cultured hemocytes from *V. parahaemolyticus*–infected mud crab treated with exosome-PBS, exosome-Vp, exosome-Vp, and DFO or Fe^2+^. *G*, the FA content in the hemocytes of *V. parahaemolyticus*–infected mud crabs treated with exosome-PBS, exosome-Vp, exosome-Vp and DFO, or Fe^2+^ was measured. *A*–*G*, data are shown as the means ± SD. Three independent repeats were performed (≥5 crabs per sample), with different lowercase letters indicating the significance (one-way ANOVA), ∗*p* < 0.05, ∗∗*p* < 0.01, ∗∗∗*p* < 0.001, ∗∗∗∗*p* < 0.0001. CD36, cluster of differentiation 36; DFO, deferoxamine; FA, fatty acid; HETE, hydroxyeicosatetraenoic acid; PPAR, peroxisome proliferator-activated receptor.

To gain insights into the promotion of ferroptosis by exosomes, we evaluated the impact of exosome-Vp and exosome-PBS on cellular 20-HETE levels. The results demonstrated that exosome-PBS treatment resulted in increased levels of 20-HETE in hemocytes (Fig. 8*A*). Additionally, treatment with exosome-Vp significantly elevated the levels of cellular Fe^2+^ (Fig. 8*B*).

We hypothesized that exosomes could modulate iron levels and thereby impact the CYP–CD36 axis, leading to enhanced ferroptosis. To test this hypothesis, we quantified the concentration of 20-HETE in *in vitro*–cultured *V. parahaemolyticus*–infected hemocytes, treated with a medium containing exosome-PBS, exosome-Vp, exosome-Vp, and deferoxamine mesylate salt (DFO, an Fe^2+^ chelator that alleviates ferroptotic cell death) or Fe^2+^. We observed a significant difference between the exosome-Vp and the DFO along with exosome-Vp–treated group (Fig. 8*C*). Subsequent experiments were conducted also using above treatment. Likewise, DFO was able to attenuate the significant increase in nuclear PPAR levels, CD36 expression, and FA content following exosome-Vp treatment (Fig. 8, *D*–*G*). Collectively, these results indicate that exosome-Vp promotes cellular ferroptosis by modulating the CYP–CD36 axis through an increase in Fe^2+^ content.

### Exosomal STEAP4 induce ferroptosis in hemocytes

**Figure 9:**
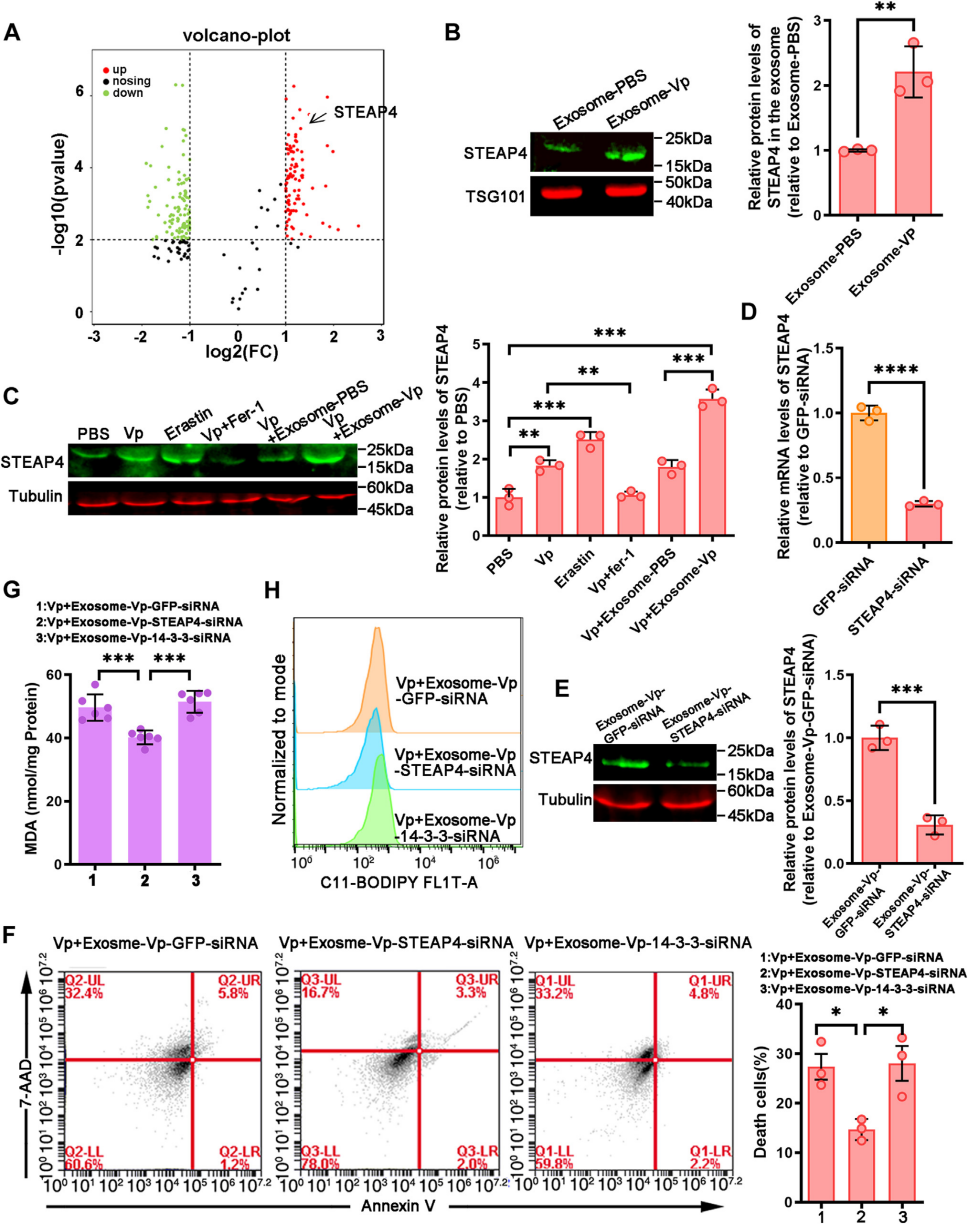
**Exosomal STEAP4 induce ferroptosis in hemocytes.***A*, protein screening identified STEAP4 as upregulated protein in exosome-Vp. *B*, STEAP4 content of exosome-PBS and exosome-Vp were detected by Western blot. *C*, Western bolt analysis of STEAP4 expression in PBS, *Vibrio parahaemolyticus*, Erastin, *V. parahaemolyticus* and Fer-1, *V. parahaemolyticus* and exosome-PBS, or *V. parahaemolyticus* and exosome-Vp–treated mud crab. *D* and *E*, the impact of STEAP4-siRNA on STEAP4 expression in mud crab hemocytes and exosome was determined using qRT-PCR and Western blot. *F*, mud crabs infected with *V. parahaemolyticus* were treated with exosome-Vp-GFP-siRNA, exosome-Vp-STEAP4-siRNA, or exosome-Vp-14-3-3-siRNA and the hemocytes were collected for annexin V/7-AAD staining. Flow cytometry was used to determine the proportion of 7-AAD–positive cells (dead cells). *G*, mud crabs infected with *V. parahaemolyticus* were treated with exosome-Vp-GFP-siRNA, exosome-Vp-STEAP4-siRNA, or exosome-Vp-14-3-3-siRNA and the level of lipid peroxidation in their hemocytes was assessed by measuring MDA levels. *H*, mud crabs infected with *V. parahaemolyticus* were treated with exosome-Vp-GFP-siRNA, exosome-Vp-STEAP4-siRNA, or exosome-Vp-14-3-3-siRNA, and the hemocytes were loaded with BODIPY 581/591 to analyze lipid ROS generation, and the fluorescent signal was detected using flow cytometry. *C*, *F* and *G*, data represents mean ± SD from three independent repeats with error bars representing S.D. The data were subjected to one-way ANOVA analysis (∗*p* < 0.05, ∗∗*p* < 0.01, ∗∗∗*p* < 0.001, ∗∗∗∗*p* < 0.0001). *B*, *D* and *E*, data represent mean ± S.D. from three independent repeats with error bars representing S.D., and the asterisk represents a significant difference calculated by the *t* test for unpaired samples. MDA, malondialdehyde; ROS, reactive oxygen species; STEAP4, six-transmembrane epithelial antigen of prostate 4.

Subsequently, we investigated the key components of exosomes. Recent studies have highlighted the crucial roles of exosomal proteins in antibacterial immunity. Consequently, we identified significant proteins present in hemolymph exosomes (23). We constructed a volcano plot to identify proteins that exhibited significant differential expression, using a moderate-to-high expression threshold. This allowed for further exploration of the underlying molecular mechanisms. Subsequently, we focused on Fe^2+^ metabolism to facilitate the interpretation of the aforementioned results. In doing so, we identified the presence of STEAP4 (Fig. 9*A*), known for its role in mediating iron metabolism. Importantly, elevated levels of STEAP4 were found in exosome-Vp compared to exosome-PBS (Fig. 9*B*). Furthermore, Western blot analysis demonstrated that both Erastin and *V. parahaemolyticus* significantly upregulated the expression of STEAP4. Additionally, Fer-1 effectively inhibited the *V. parahaemolyticus*–induced STEAP4 expression, while exosome-Vp further enhanced the expression of STEAP4 induced by *V. parahaemolyticus*, indicating a potential role for STEAP4 in ferroptosis (Fig. 9*C*).

To investigate the relationship between the induction of ferroptosis by exosome-Vp and STEAP4, we performed a knockdown of STEAP4 in mud crab hemocytes using STEAP4-siRNA injection (Fig. 9*D*). After injecting GFP-siRNA, STEAP4-siRNA, and 14-3-3-siRNA (another protein that upregulated in exosome) into the hemolymph of *V. parahaemolyticus*–infected mud crab (23), exosomes (exosome-Vp-GFP-siRNA, exosome-Vp-STEAP4-siRNA, and exosome-Vp-14-3-3-siRNA) were extracted and Western blot analysis was performed to detect the STEAP4 content in exosomes (Fig. 9*E*). Flow cytometry analysis of cell death demonstrated that exosome-Vp-GFP-siRNA and exosome-Vp-14-3-3-siRNA were found to increase the number of ferroptotic hemocytes in *V. parahaemolyticus*–infected mud crab compared with exosome-Vp-STEAP4-siRNA, which indicated exosomes with lower levels of STEAP4 had a smaller effect on ferroptosis in mud crab hemocytes. (Fig. 9*F*). Additionally, the content of STEAP4 in exosomes significantly influences its impact on lipid peroxidation. (Fig. 9, *G* and *H*). Collectively, the results indicate that exosome-Vp regulates hemocyte ferroptosis through interaction with STEAP4 in the mud crab.

### Exosomal STEAP4 induce hemocytes ferroptosis *via* CYP–CD36 axis

**Figure 10:**
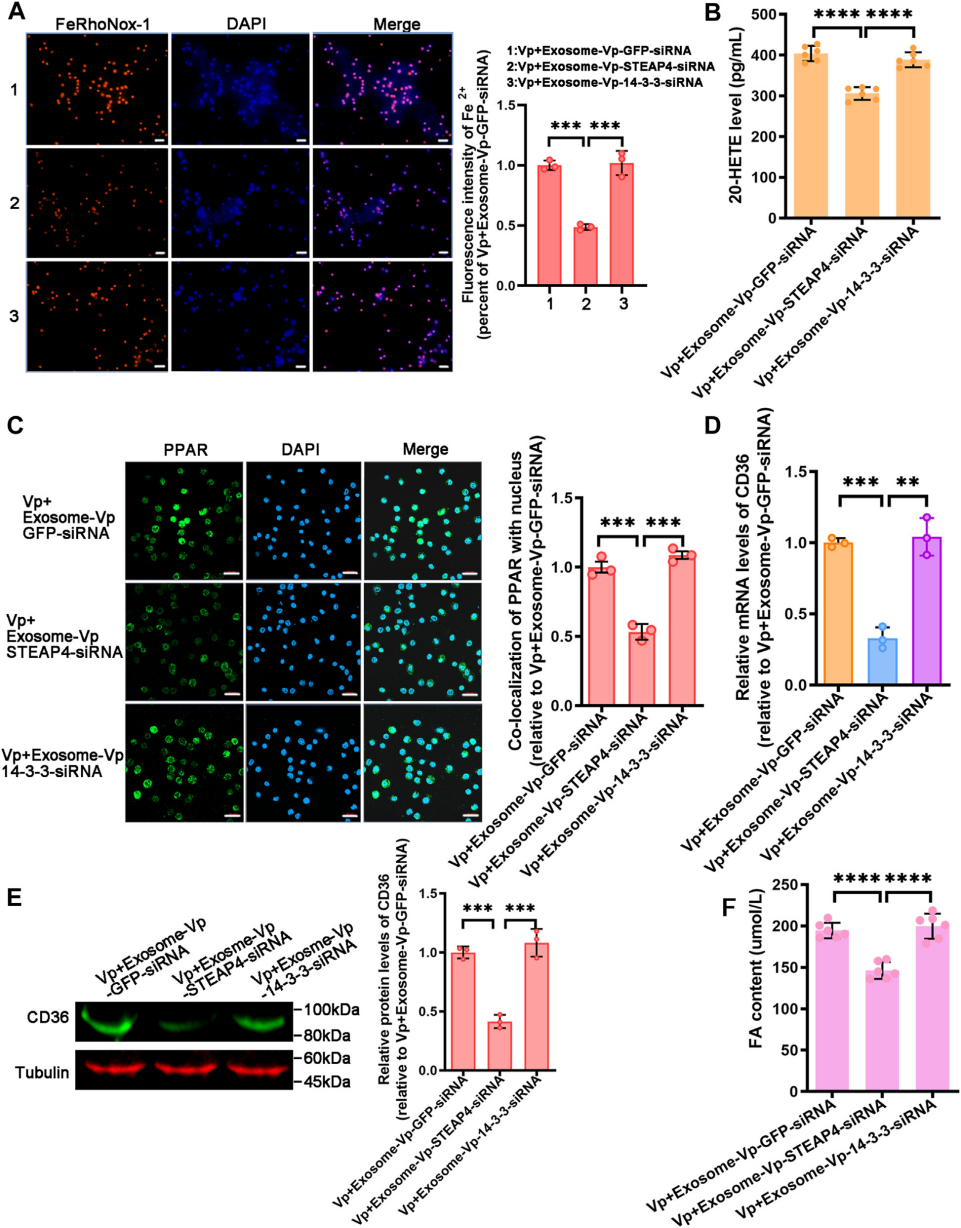
**Exosomal STEAP4 induce hemocytes ferroptosis *via* CYP/CD36 axis.***A*, Fe^2+^ levels in mud crabs infected with *Vibrio parahaemolyticus* and subsequently treated with exosome-Vp-GFP-siRNA, exosome-Vp-STEAP4-siRNA, or exosome-Vp-14-3-3-siRNA were measured using confocal microscopy with FeRhoNox-1 staining. Scale bars represent 50 μm. *B*, the generation of 20-HETE was detected in mud crabs infected with *V. parahaemolyticus* followed by treatment with exosome-Vp-GFP-siRNA, exosome-Vp-STEAP4-siRNA, or exosome-Vp-14-3-3-siRNA. *C*, the translocation of PPAR in mud crabs infected with *V. parahaemolyticus* was analyzed using confocal microscopy after treatment with exosome-Vp-GFP-siRNA, exosome-Vp-STEAP4-siRNA, or exosome-Vp-14-3-3-siRNA. Scale bars represent 20 μm. *D* and *E*, the expression of CD36 in mud crabs infected with *V. parahaemolyticus* was analyzed by qRT-PCR and Western blot after treatment with exosome-Vp-GFP-siRNA, exosome-Vp-STEAP4-siRNA, or exosome-Vp-14-3-3-siRNA. *F*, the FA content was measured in mud crabs infected with *V. parahaemolyticus* and treated with exosome-Vp-GFP-siRNA, exosome-Vp-STEAP4-siRNA, or exosome-Vp-14-3-3-siRNA. Data are shown as the means ± SD. Three or six independent repeats were performed (≥5 crabs per sample), with different lowercase letters indicating the significance (one-way ANOVA), ∗∗*p* < 0.01, ∗∗∗*p* < 0.001, ∗∗∗∗*p* < 0.0001. CD36, cluster of differentiation 36; FA, fatty acid; HETE, hydroxyeicosatetraenoic acid; PPAR, peroxisome proliferator-activated receptor; STEAP4, six-transmembrane epithelial antigen of prostate 4.

STEAP4, a metalloreductase involved in iron homeostasis, is believed to play a pivotal role in ferroptosis by modulating iron metabolism. To confirm this hypothesis, we used FeRhoNox-1 staining and confocal microscopy to measure the levels of Fe^2+^. The results demonstrated that the content of STEAP4 in exosomes affects its regulation of Fe^2+^ levels of hemocytes (Fig. 10*A*). These findings strongly proved that exosomal STEAP4 plays a critical role in increasing intracellular Fe^2+^ levels in *V. parahaemolyticus*–infected mud crabs.

Since CYP is a major source of catalytic iron, it remains unclear whether exosome-Vp promotes ferroptosis by increasing intracellular Fe^2+^ through STEAP4, thereby inducing lipid ROS and upregulating CYP activity. Subsequently, we analyzed the levels 20-HETE of hemocytes and its downstream molecules, PPAR and CD36, which are involved in FA accumulation. The results demonstrated that the content of STEAP4 in exosomes had significant impact on the levels of 20-HETE and the translocation of PPAR from the cytoplasm to the nucleus (Fig. 10, *B* and *C*). Likewise, Exosome-Vp-GFP-siRNA and exosome-Vp-14-3-3-siRNA promoted the expression of CD36, while exosomes with lower levels of STEAP4 (exosome-Vp-STEAP4-siRNA) exhibit a reduced impact on CD36 expression. (Fig. 10, *D* and *E*). Importantly, the impact of exosomes with lower levels of STEAP4 on the accumulation of FAs in hemocytes was also diminished (Fig. 10*F*). These findings show that exosomal STEAP4 plays a crucial role in regulating Fe^2+^ levels, thereby facilitating the CYP–CD36 axis and influencing hemocyte ferroptosis.

## Discussion

Ferroptosis is a regulated form of cell death that relies on iron and has recently been recognized as an intriguing host defense mechanism against microbial invasions. In this study, we investigated the role of exosomes in driving ferroptosis and inhibiting bacterial infection in mud crab, shedding some lights on the immune response mechanisms employed by crustaceans.

Our findings have unveiled the vital role of exosomes in the immune defense of crustaceans. Exosomes can serve as an innate immune response to bacterial infection (29). Decoy exosomes offer protection against toxins produced by *S. aureus* in both human cells and mice (30). Exosome-mediated antibacterial immunity also represents a general antibacterial strategy in crustaceans. During bacterial infection, exosomal miR-224 contributes to hemolymph microbiota homeostasis, while exosomal 14-3-3 inhibits bacterial infection by activating anti-lipopolysaccharide factors in mud crabs (23, 25). Recent studies have indicated that exosomes regulate ferroptosis by delivering biological material to recipient cells, influencing ferroptosis-related proteins or transporting ferritin-bound iron out of cells (31, 32). This study revealed that exosomes play a crucial role in orchestrating the immune response and combating microbial infections by promoting intracellular iron accumulation and activating ferroptotic pathways.

Ferroptosis is triggered by Fe^2+^ through Fenton reactions and the accumulation of ROS (33). Exosomes can influence ferroptosis by participating in iron metabolism (28). Exosomal miR-30b-5p plays a crucial role in ferroptosis by downregulating the expression of ferroportin 1 (an iron exporter), leading to increased levels of labile Fe^2+^ (34). Furthermore, exosomes can directly transport iron out of the cell to inhibit ferroptosis (35). Enzymes belonging to the STEAP family aid in the uptake of metal ions by mammalian cells by reducing Fe^3+^ and Cu^2+^ (36). By inhibiting STEAP3), exosomal miR-124-3p exerts an inhibitory effect on ferroptosis (37). This study identified STEAP4 protein within exosomes as a crucial factor that boosts cellular Fe^2+^ levels. The rise in intracellular iron levels triggers Fenton reactions, resulting in the production of highly reactive hydroxyl radicals through the interaction between iron and hydrogen peroxide. This pathway underscores the complex interplay among exosomes, iron metabolism, and ferroptosis in the immune defense of crustaceans.

Furthermore, we have discovered an additional mechanism by which exosomes contribute to the induction of ferroptosis. The accumulation of lipid peroxides is considered a determinant in the occurrence of ferroptosis (38). Lipid peroxides are directly regulated by oxygenases, such as lipoxygenases, CYP, and prostaglandin-endoperoxide synthase. CYP triggers ferroptosis by converting dihomo-γ-linolenic acid to dihydroxyeicosadienoic acid (39). In our study, we found that CYP regulates the expression of CD36, the fatty acid receptor, to induce ferroptosis. Moreover, CYP plays a critical role in the production of 20-HETE, the omega-hydroxylation product of AA catalyzed by CYP enzymes, which can activate PPAR (40, 41). Inhibition of selective 20-HETE synthase could reduce STING-induced ferroptosis in peripheral blood mononuclear cells from human and murine subjects with sepsis (42). Ferroptosis is promoted by MDM2 and MDMX through PPARα-mediated lipid remodeling (43). CD36, a scavenger receptor regulated by PPAR, can mediate fatty acid uptake, leading to lipid peroxidation and the induction of ferroptosis (44). Upon activation, PPAR induces the upregulation of CD36, a downstream target gene. CD36, a scavenger receptor for fatty acids, plays a crucial role in facilitating the buildup of fatty acids and promoting lipid peroxidation, ultimately resulting in ferroptosis. This exosomal pathway emphasizes the complex regulatory network associated with immune defense and ferroptosis.

Currently, the involvement of exosomal pathways in ferroptosis remains poorly understood. Here, this study demonstrates that exosomes play a role in promoting ferroptosis in invertebrate mud crab by mediating the CYP–CD36 pathway through carrying STEAP4. In vertebrates, it has been observed that exosomes can inhibit ferroptosis either by directly removing iron from cells or by reducing the expression of iron exporters (28, 34). Moreover, miR-124-3p in heme oxygenase oxygen-1–modified bone marrow mesenchymal stem cells–derived exosomes downregulates STEAP3 expression to inhibit ferroptosis, thereby attenuating graft ischemia-reperfusion injury (37). It should be noted that the possibility of exosomes regulating ferroptosis through STEAP family proteins in vertebrates cannot be dismissed. Furthermore, the involvement of the CYP–CD36 axis in ferroptosis is supported to some extent by evidence suggesting that metabolites of polyunsaturated fatty acids from the cytochrome P450 and epoxide hydrolase pathways can modulate neurodegeneration *via* ferroptosis (39). Additionally, CD36 activity has been found to induce ferroptosis in tumor-infiltrating CD8+ T cells (44). However, further experimental studies are required to verify the role of the CYP–CD36 axis in ferroptosis in other species. Although the ferroptosis pathway was not completely understood in vertebrates, the exosome-mediated ferroptosis elucidated in invertebrates mud crab might be also existed in vertebrates. In this study, the novel mechanism of which exosomes regulate ferroptosis would be universal from invertebrates to vertebrates, thus providing some valuable insights into the pathogenesis of related diseases, offering potential targets for future therapeutic strategies.

**Figure 11:**
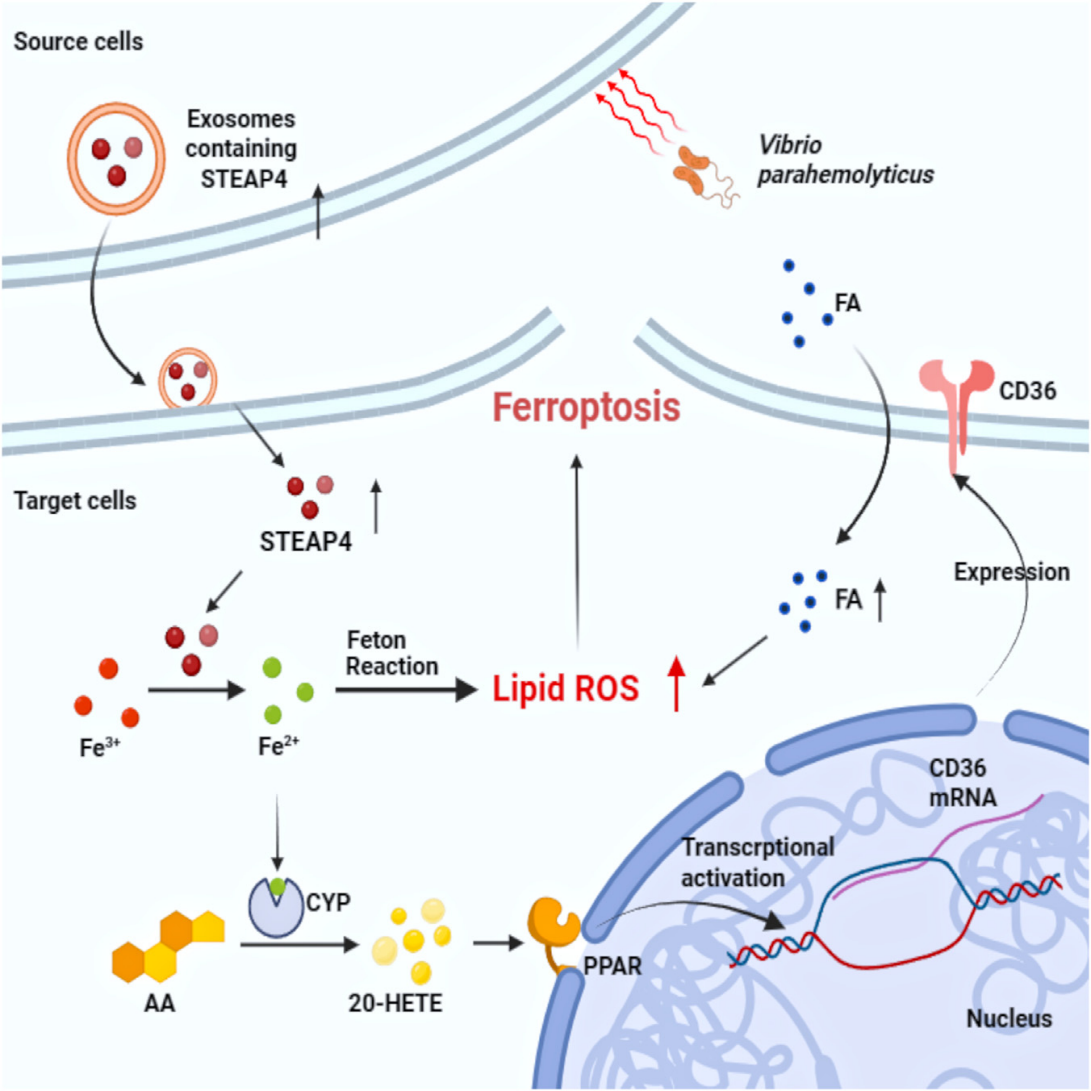
**Exosomes drive ferroptosis by stimulating iron accumulation to inhibit bacterial infection in crustaceans.** After bacterial infection, there is more packaging of STEAP4 in the mud crab exosomes. Then, this increased uptake of exosomal STEAP4 enhances cellular Fe^2+^ levels, triggering Fenton reactions and activation of CYP in hemocytes. The iron-dependent enzyme CYP leads more 20-HETE synthesized from arachidonic acid. 20-HETE can promote translocation of PPAR from the cytoplasm into the nucleus to regulate the expression of CD36. Finally, CD36 promotes fatty acid accumulation, lipid peroxidation, and ferroptosis. CD36, cluster of differentiation 36; HETE, hydroxyeicosatetraenoic acid; PPAR, peroxisome proliferator-activated receptor; STEAP4, six-transmembrane epithelial antigen of prostate 4.

In conclusion, the hemolymph exosomes transport a higher amount of STEAP4 into the hemocytes during bacterial infection. Exosomal STEAP4 further enhances ferroptosis by facilitating the accumulation of Fe^2+^. The interaction between Fe^2+^ and H_2_O_2_ in the Fenton reaction generates hydroxyl radicals, leading to oxidative damage to DNA, proteins, and membrane lipids, ultimately inducing ferroptosis. Moreover, Fe^2+^ promotes CYP metabolism of AA, resulting in the production of 20-HETE, which triggers the translocation of PPAR from the cytoplasm to the nucleus. Consequently, PPAR activates the transcription of CD36, which regulates fatty acid uptake and induces ferroptosis in hemocytes (Fig. 11). This study highlights the potential of exosomes in protecting crustaceans against bacterial infections. These findings not only advance our understanding of host defense mechanisms in crustaceans but also provide potential strategies for managing bacterial infections in crustaceans. Continued research in this field has the capacity to revolutionize our approach to combating microbial infections and promoting strategies in ferroptosis-related therapy in some diseases.

## Experimental procedures

### Ethics statement

Animals used in this study were approved by the Guangdong Provincial Department of Science and Technology on the Use and Care of Animals and did not involve endangered or protected species. The animals were processed according to “the Regulations for the Administration of Affairs Concerning Experimental Animals” established by the Guangdong Provincial Department of Science and Technology on the Use and Care of Animals.

### Animals and primary cultured hemocytes

Healthy mud crabs weighing approximately 50 g each were obtained from a local crab farm located in Niutianyang, Shantou, Guangdong, China. They were acclimated to laboratory conditions with a salinity of 8% and a temperature of 25 °C for 1 week prior to subsequent procedures.

The primary culture of mud crab hemocytes was performed using established techniques. Hemolymph was collected by extracting it from the nonsclerotized membrane of the posterior walking leg. A sterile syringe with a 10 ml capacity, preloaded with 5 ml of precooled sterile anticoagulant solution (consisting of 0.14 M NaCl, 0.1 M glucose, 30 mM trisodium citrate, 26 mM citric acid, 10 mM EDTA, and adjusted to pH 4.6), was utilized for this purpose. The hemolymph and anticoagulant were mixed in a 1:1 ratio. Subsequently, the hemolymph was immediately centrifuged at 800*g* and 4 °C for 10 min. Following centrifugation, the supernatant was discarded, and the pellet was washed with PBS. The obtained hemocytes were gently resuspended in pure Leibovitz's L-15 medium (Sigma-Aldrich), supplemented with 1% antibiotics (10,000 units/ml penicillin and 10,000 μg/ml streptomycin from Gibco), and 0.2 mM NaCl (with an osmolarity of 676 ± 5.22 mOsm/kg). The pH of the medium was adjusted to a range of 7.2 to 7.4. The hemocytes were then quantified using an automated cell counter (Thermo Fisher Scientific). Subsequently, 4 ml of the hemocyte suspension, containing a density of 1 × 10^6^ cells/ml, was plated in 60-mm dishes without any additional additives.

### Bacterial challenge and sample collection

*V. parahaemolyticus* were obtained from the National Pathogen Collection Center for Aquatic Animals (nos. BYK00036). *V. parahaemolyticus* is one of the most dangerous pathogens for aquatic animals. The bacteria were cultured, collected, and resuspended in sterile PBS (137 mM NaCl, 2.7 mM KCl, 10 mM Na_2_HPO_4_, 2 mM KH_2_PO_4_, and pH 7.4). Bacterial counts were determined using the agar plating method.

*In vitro* and *in vivo* bacterial challenges were conducted according to a previously described method. For *in vitro* bacterial stimulation, cultured bacteria (1 × 10^7^ cells per dish, 50 μl) suspended in sterile PBS (50 μl) were separately added to a dish containing hemocytes as the control. Hemocytes were collected at specific time points following bacterial stimulation for total RNA extraction. Each sample consisted of at least three crabs. Reverse transcription of first-strand cDNA was performed using a Reverse Transcriptase Kit (Cat: KR118; TIANGEN) in accordance with the manufacturer's instructions. For *in vivo* bacterial infection, bacteria (1 × 10^6^ colony-forming units per crab, 200 μl) were injected into the hemolymph from a nonsclerotized membrane of a posterior walking leg different from the site used for hemolymph collection. As a control, sterile PBS (200 μl) was injected instead. Total RNA was extracted using TRIzol reagent (Cat: 15596026; Thermo Fisher Scientific). Cytoplasmic and nuclear proteins were extracted separately using the NE-PER kit (Cat: 78835; Thermo Fisher Scientific).

### RNA interference assay

Based on the sequence of CYP (GenBank accession number: MN782363.1), STEAP4 (OR050525), CD36 (OR050526), and PPAR (OR050527), the siRNA specifically targeting gene was designed (Table S1). The siRNA was synthesized using the *In vitro* Transcription T7 Kit (Cat: 6104; TaKaRa) according to the manufacturer’s instructions. Subsequently, 25 μg of CYP-siRNA, STEAP4-siRNA, CD36-siRNA, and PPAR-siRNA were individually injected into each mud crab. To enhance the efficiency of RNAi, a second injection was performed 24 h after the initial one. For control purposes, siGFP was utilized. Hemocytes were collected from the mud crabs at 24 h following the second injection, and total RNA was extracted. The expressions of these genes were analyzed using RT-QPCR with primers (RT-F and RT-R) (Table S1) to evaluate the effectiveness of RNAi. All experiments were conducted in triplicate.

### Bacteria clearance and survival rate assays

Following the knockdown of CYP, STEAP4, CD36, and PPAR using RNAi, each crab received an injection of 200 μl exosome solution (1 × 10^8^ vesicles/ml) or *V. parahaemolyticus* (1 × 10^6^ cfu/ml) at the base of the fourth leg. After 48 h, the hemolymph was collected, diluted, and spread onto 2216E agar plates for overnight incubation at 37 °C. The subsequent bacterial colonies were then counted. The cumulative mortality of mud crabs was monitored daily for a period of 72 h. All experiments were conducted in triplicate. The obtained data were subjected to statistical analyses.

### Isolation and analysis of exosomes

To isolate exosomes, the hemolymph of mud crabs was collected and subjected to centrifugation at 800*g* for 5 min to obtain the supernatants. The supernatants were then subjected to ultracentrifugation, followed by sucrose density-gradient centrifugation. The solution between 1.3 M and 0.95 M sucrose concentration was collected and filtered through 0.22 μm filters. The resulting exosomes were visualized using a Philips CM120 BioTwin transmission electron microscope (FEI Company) and quantified using Nano-Sight NS300 (Malvern Instruments Ltd).

### Immunoprecipitation analysis

The hemolymph was collected from three mud crabs and immediately subjected to centrifugation at 800*g* to isolate the hemocytes. Subsequently, the hemocytes were washed with PBS and fixed by adding 4% paraformaldehyde. Following a 10-min incubation in 0.1% Triton X-100, the hemocytes were blocked with 5% bovine serum albumin for 40 min. Overnight, the hemocytes were incubated with specific antibodies. After washing with PBS (140 mM NaCl, 2.7 mM KCl, 10 mM Na_2_HPO_4_, 1.8 mM KH_2_PO_4_, pH 7.4), the hemocytes were incubated with the secondary antibody, goat anti-rabbit Alexa Fluor 488 (diluted 1:1000 in 1% bovine serum albumin). The reaction was then kept in the dark for 1 h before washing with PBS. The hemocyte nucleus was stained with DAPI for 10 min at room temperature and subsequently washed again. Finally, the hemocytes were observed under a fluorescence microscope (Olympus IX71).

### Assessment of lipid peroxidation

Hemocytes were incubated with 2 μM C11-BODIPY581/591 (a marker for lipid peroxidation) obtained from Thermo Fisher Scientific (Cat:D3861) for 30 min at 37 °C in the absence of light. Following the loading period, any unincorporated dye was eliminated by washing with PBS containing 2% FBS. Subsequently, the samples were centrifuged at 1000 rpm for 3 min, and the resulting pellets were resuspended in 500 μl of PBS containing 2% FBS. Flow cytometry measurements were carried out using a FACSCalibur flow cytometer (Becton Dickinson). The fluorescence intensity of each probe was quantified using the FlowJo software program (http://www.flowjo.com). Plots displaying the mean percentages ± SD of positive cells relative to the total cell population are depicted.

### Measurement of GSH

Total glutathione in hemocyte lysates or tissue lysates were measured with GSH detection kit (Cat: S0053; Beyotime) according to the manufacturer’s instruction.

### Measurement of MDA

The hemocyte lysates were measured with MDA detection kit (Cat: S0131S; Beyotime) according to the manufacturer’s instruction.

### Intracellular Fe^2+^ imaging by fluorescence microscopy

FeRhonox-1 (Cat: HY-D1533; MCE), a commercial fluorescent probe that specifically binds Fe^2+^, was used to determine intracellular Fe^2+^ content. The hemocytes in different treatment were immobilized with 4% paraformaldehyde for 1 h and washed thrice with PBS. After the addition of 5 μM FeRhonox-1, stained cells were placed in the dark for 20 min. Then, Fe^2+^ images were obtained using a fluorescence microscope (Olympus IX71).

### Cell death analysis

Cell death analysis of ferroptosis followed the method described by Chen *et al.* (45). After treatments, cells were collected and stained with Annexin V-FITC/7-AAD Apoptosis Kit (Cat: KA3806; Abnova) and subjected to flow cytometry. The percentages of dead cells were quantified with CellQuest software (https://www.bdbiosciences.com/zh-cn/products/instruments/software-informatics/instrument-software/bd-sce-software-templates-for-bd-cellquest-and-bd-cellquest-pro.648089).

### Calcein-AM/PI fluorescence staining

After the indicated treatment, hemocytes were washed twice with PBS, and a solution of 2 μm/l calcein-AM and 4.5 μm/l PI (Cat: 04511; Sigma) was added to the culture wells and incubated at 37 °C in the dark for 20 min. Living cells (green cytoplasmic fluorescence) and dead cells (red nucleus) were observed in an inverted fluorescence microscope (Olympus IX71). Live:dead cell ratio from ten random fields was quantified with ImageJ software (https://imagej.net/software/imagej).

### Constitutive active PPAR plasmid construction

The constitutive active PPAR plasmid construction followed the method described by Wang *et al.* (26). A PCR fragment representing the mature PPAR was amplified using specific primers (Table S1). Subsequently, the PCR products were purified, digested with restriction enzymes, and ligated into the pAdlox vector (BioVector). The recombinant plasmid pAdlox-PPAR was transformed into primary cultured hemocytes by Lipofectamine 3000 (Thermo Fisher Scientific).

### Western blot analysis

Protein mixed with 5× SDS sample buffer and separated by 12% SDS-PAGE. The proteins in the gel were transferred onto a nitrocellulose membrane, followed by blocking with 5% nonfat milk dissolved in TBST and incubated with 1:100 diluted antiserum at 4 °C overnight. The membrane was washed with TBST and incubated with Goat Anti-Mouse IgG H&L (Alexa Fluor 647) or Goat Anti-Rabbit IgG H&L (Alexa Fluor 647) and subsequently detected by Invitrogen iBright FL1500 (Thermo Fisher Scientific). The primary antibody of tubulin, CYP, STEAP4, CD36, and PPAR antibodies used were mouse polyclonal antibodies prepared in our lab and TSG101 (Fig. S1, *A*–*E*).

## Data availability

All data are contained within the article.

## Supporting information

This article contains supporting information.

## Conflict of interest

The authors declare that they have no conflicts of interest with the contents of this article.
