# Supplementary material for: Exosomes drive ferroptosis by stimulating iron accumulation to inhibit bacterial infection in crustaceans
Source: J Biol Chem. 2023 Nov 15;299(12):105463. doi: 10.1016/j.jbc.2023.105463 (PMC10704439; doi:10.1016/j.jbc.2023.105463)
Supplement: Supporting Table S1 [file mmc1.docx]

**Supplementary Information Table**

**Exosomes drive ferroptosis by stimulating iron accumulation**

**to inhibit bacterial infection in** **crustaceans**

Qian Sun^1,2,3^, Jiawen Yang^1,2,3^, Ming Zhang^1,2,3^, Yongsheng Zhang^1,2,3^, Hongyu Ma^1,2,3^, Ngoc Tuan Tran^1,2,3^, Xiuli Chen^4^, Yueling Zhang^1,2,3^, Kok-Gan Chan^2,5^, Shengkang Li^1,2,3^*

Table S1. Primers and oligonucleotides used in this study

| Name | Sequence | Purpose |
| --- | --- | --- |
| CD36-F | GTAGTGGCTTCTCCG | qPCR |
| CD36-R | TGTTGTATGCCTTTGA |  |
| siCD36-1 | GATCACTAATACGACTCACTATAGGGGCCCTTGAGTTTGCCTTCAAGAGTGTT | RNAi |
| siCD36-2 | AACACTCTTGAAGGCAAACTCAAGGGCCCCTATAGTGAGTCGTATTAGTGATC |  |
| siCD36-3 | AAGCCCTTGAGTTTGCCTTCAAGAGTGCCCTATAGTGAGTCGTATTAGTGATC |  |
| siCD36-4 | GATCACTAATACGACTCACTATAGGGCACTCTTGAAGGCAAACTCAAGGGCTT |  |
| STEAP-F | TCCAGGGGCCCCTGGATGGTGGTGGTGGCCGTGGGGC | PCR screening |
| STEAP-R | CGCTCGAGTCGACCCTCACGTCGAGATTGACAGTATG |  |
| Q-STEAP4-F | GCACTACCGCAGCAAC | qPCR |
| Q-STEAP4-R | CACCATCTCGGATACCTT |  |
| siSTEAP4-1 | GATCACTAATACGACTCACTATAGGGGCGGTTGTGTTTGGCATCTTCGTCTTT | RNAi |
| siSTEAP4-2 | AAAGACGAAGATGCCAAACACAACCGCCCCTATAGTGAGTCGTATTAGTGATC |  |
| siSTEAP4-3 | AAGCGGTTGTGTTTGGCATCTTCGTCTCCCTATAGTGAGTCGTATTAGTGATC |  |
| siSTEAP4-4 | GATCACTAATACGACTCACTATAGGGAGACGAAGATGCCAAACACAACCGCTT |  |
| Q-COX2-F | TTTTCGTGAGACTTTCCT | qPCR |
| Q-COX2-R | TATCGGCAGTATGACCAG |  |
| Q-ACSL4-F | TCGCCCTCATCTCCCT | qPCR |
| Q-ACSL4-R | GCTGCTCCACCTTCTGC |  |
| Q-PTGS2-F | CTGGATGAGGGAGCACAA | qPCR |
| Q-PTGS2-R | ATAATGAGGCGGGCAGTC |  |
| Q-NOX-F | CCGCACAATAGTGGTGAAAT | qPCR |
| Q-NOX-R | CTCTGCGATGGGCACTAACC |  |
| Q-GPX4-F | CTGCTGGTCCTTCCTTCT | qPCR |
| Q-GPX4-R | TCTTGCCTCATTTGCTTG |  |
| Q-FTH1-F | CGCCAGAACTACCACCAG | qPCR |
| Q-FTH1-R | TTCAAAGCCACATCATCG |  |
| CYP-F | TCCAGGGGCCCCTGGATGGGCGTGGAGGTGTGGGTGT | PCR screening |
| CYP-R | TCACGATGCGGCCGCTTATTCTGCCACGATCGGCTTT |  |
| Q-CYP-F | TGTGCGACAAGGCTGAT | qPCR |
| Q-CYP-R | TGCTTTGCGAGGGAAT |  |
| siCYP-1 | GATCACTAATACGACTCACTATAGGGCGCCTTTCCTCGGACACATACTGAATT | RNAi |
| siCYP-2 | AATTCAGTATGTGTCCGAGGAAAGGCGCCCTATAGTGAGTCGTATTAGTGATC |  |
| siCYP-3 | AACGCCTTTCCTCGGACACATACTGAACCCTATAGTGAGTCGTATTAGTGATC |  |
| siCYP-4 | GATCACTAATACGACTCACTATAGGGTTCAGTATGTGTCCGAGGAAAGGCGTT |  |

**Supplementary Table 1.** The sequence of primers used in the study. Related to Figure STAR method.
