# Supplementary material for: Exosomes drive ferroptosis by stimulating iron accumulation to inhibit bacterial infection in crustaceans
Source: J Biol Chem. 2023 Nov 15;299(12):105463. doi: 10.1016/j.jbc.2023.105463 (PMC10704439; doi:10.1016/j.jbc.2023.105463)
Supplement: Supporting Figures S1 and S2 [file mmc2.pdf]

Supplementary Information Figures

Exosomes drive ferroptosis by stimulating iron accumulation to inhibit bacterial infection in crustaceans

Qian Sun[1,2,3], Jiawen Yang[1,2,3], Ming Zhang[1,2,3], Yongsheng Zhang[1,2,3], Hongyu Ma[1,2,3], Ngoc Tuan Tran[1,2,3], Xiuli Chen[4], Yueling Zhang[1,2,3], Kok-Gan Chan[2,5], Shengkang Li[1,2,3]*

Figure S1

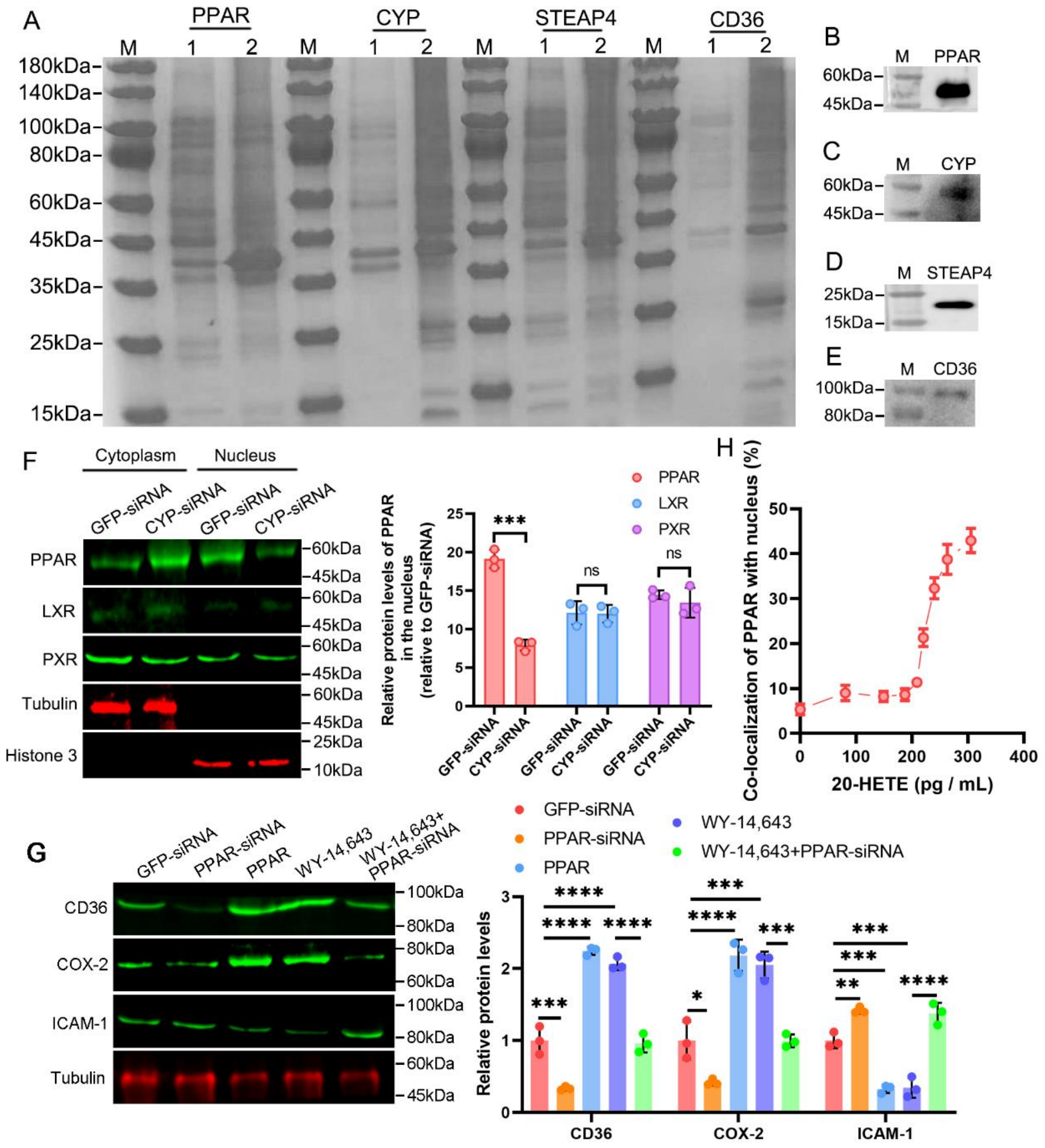

(A) Recombinant expression of PPAR-pGET28a, CYP-pGET28a, STEAP4-pGET28a, CD36-pGET28a in *E. coli*. lane M, protein molecular mass marker. lane 1, total proteins from *E. coli* without IPTG induction; lane 2, total proteins from *E. coli* with IPTG induction; (B), (C), (D), (E) The recombinant protein tested by western blotting with special antibody; (F) Nuclear and cytoplasmic proteins were extracted to analyze the translocation of PPAR, LXR and PXR in hemocytes of CYP-silenced mud crab; (G) The impact of PPAR on Its target genes; (H) The percentage of PPAR in nuclues was analyzed in mud crabs with indicated 20-HETE.

Figure S2

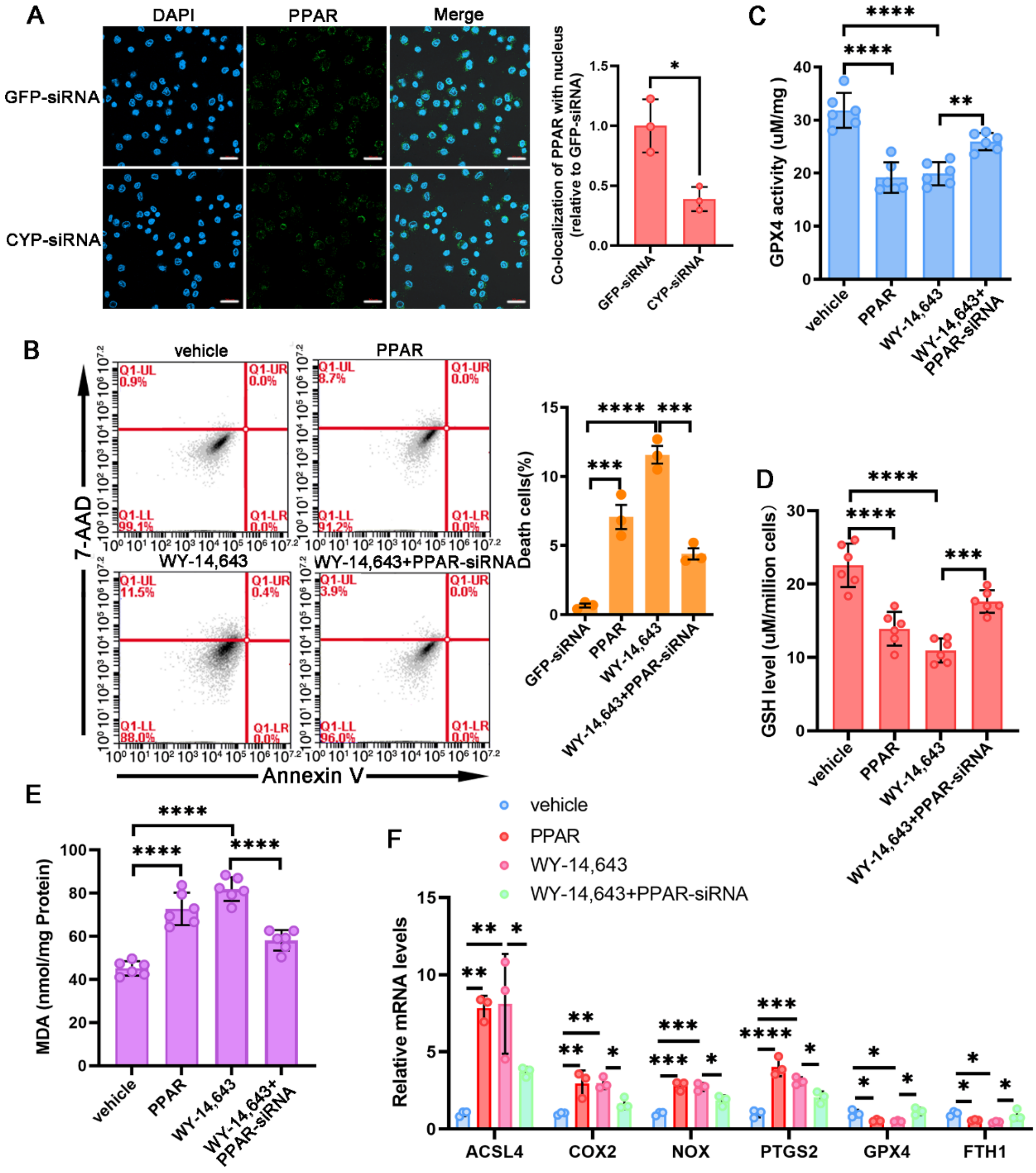

(A) Translocation of PPAR in hemocytes. *Scale bars*, 20 μm; (B) annexin V/7-AAD staining was evaluated in hemocytes; (C) GSH levels were determined using a GSH assay kit; (D) GPX4 activity were analyzed using a GPX4 assay kit; (E) MDA levels were measured in hemocytes; (F) qRT-PCR was used to check the expression of ferroptosis-related genes.
